# Anti-EMT properties of CoQ0 attributed to PI3K/AKT/NFKB/MMP-9 signaling pathway through ROS-mediated apoptosis

**DOI:** 10.1186/s13046-019-1196-x

**Published:** 2019-05-08

**Authors:** Hsin-Ling Yang, Varadharajan Thiyagarajan, Pei-Chun Shen, Dony Chacko Mathew, Kai-Yuan Lin, Jiunn-Wang Liao, You-Cheng Hseu

**Affiliations:** 1 Institute of Nutrition, College of Biopharmaceuticals and Food Sciences, China Medical University, Taichung, 40402 Taiwan; 2 Department of Cosmeceutics, College of Biopharmaceutical and Food Sciences, China Medical University, No. 91, Hsueh-Shih Road, Taichung, 40402 Taiwan; 3 Department of Medical Research, Chi-Mei Medical Center, Tainan, 710 Taiwan; 4 Graduate Institute of Veterinary Pathology, National Chung Hsing University, Taichung, 40227 Taiwan; 5 Department of Health and Nutrition Biotechnology, Asia University, Taichung, 41354 Taiwan; 6 Chinese Medicine Research Center, China Medical University, Taichung, 40402 Taiwan; 7 Research Center of Chinese Herbal Medicine, China Medical University, Taichung, 40402 Taiwan

**Keywords:** CoQ_0_, TNBC, EMT, Metastasis, Human epidermal growth factor receptor 2, TGF-β1, ROS

## Abstract

**Background:**

Breast cancer is the most prevalent cancer among women. In triple-negative breast cancer (TNBC) cells, a novel quinone derivative, coenzyme Q_0_ (CoQ_0_), promotes apoptosis and cell-cycle arrest. This study explored the anti-epithelial–mesenchymal transition (EMT) and antimetastatic attributes of CoQ_0_ in TNBC (MDA-MB-231).

**Methods:**

Invasion, as well as MTT assays were conducted. Lipofectamine RNAiMAX was used to transfect cells with β-catenin siRNA. Through Western blotting and RT-PCR, the major signaling pathways’ protein expressions were examined, and the biopsied tumor tissues underwent immunohistochemical and hematoxylin and eosin staining as well as Western blotting.

**Results:**

CoQ_0_ (0.5–2 μM) hindered tumor migration, invasion, and progression. Additionally, it caused MMP-2/− 9, uPA, uPAR, and VEGF downregulation. Furthermore, in highly metastatic MDA-MB-231 cells, TIMP-1/2 expression was subsequently upregulated and MMP-9 expression was downregulated. In addition, CoQ_0_ inhibited metastasis and EMT in TGF-β/TNF-α-stimulated non-tumorigenic MCF-10A cells. Bioluminescence imaging of MDA-MB-231 luciferase–injected live mice demonstrated that CoQ_0_ significantly inhibited metastasis of the breast cancer to the lungs and inhibited the development of tumors in MDA-MB-231 xenografted nude mice. Silencing of β-catenin with siRNA stimulated CoQ_0_-inhibited EMT. Western blotting as well as histological analysis established that CoQ0 reduced xenografted tumor development because apoptosis induction, cell-cycle inhibition, E-cadherin upregulation, β-catenin downregulation, and metastasis and EMT regulatory protein modulation were observed.

**Conclusions:**

CoQ_0_ inhibited the progression of metastasis as well as EMT (in vitro and in vivo). The described approach has potential in treating human breast cancer metastasis.

**Electronic supplementary material:**

The online version of this article (10.1186/s13046-019-1196-x) contains supplementary material, which is available to authorized users.

## Background

Worldwide, breast cancer is the commonest cancer to affect women, and in Taiwanese women, it is the leading cause of deaths from cancer [1]. It possesses highly metastatic and invasive properties, which explain its high mortality rate [2]*.* Among breast cancers, triple-negative breast cancers (TNBCs) lacking the genes for estrogen receptor, HER2, and progesterone receptor have been correlated with tumor aggressiveness. TNBCs are more likely than other breast cancer types to migrate beyond the breast and to recur after chemotherapy or lumpectomy [3]*.* TNBC cases comprise 15–20% of all breast cancer cases. Furthermore, patients with TNBC exhibit unfavorable outcomes compared with those with other breast cancer subtypes [4]. TNBC tumor cells lack the requisite receptors, which renders some targeted or hormone therapies ineffectual. Consequently, combinations of chemotherapy medicines are typically prescribed for patients with TNBC. This approach, however, does not help patients with cancer to counter the chemotherapy-induced adverse side effects and drug resistance [5]. Thus, novel compounds with lower toxicity are urgently required for effective treatment of TNBC.

In cancer cells, polarized epithelial cells complete multifaceted changes that cause them to begin expressing a mesenchymal phenotype and undergo migration, invasion, and metastasis. This process is referred to as the epithelial–mesenchymal transition (EMT) [6]. Several factors induce EMT in vitro and in vivo, for example, TGF-β1, ROS, TNF-α, and hypoxia [7–9]. EMT involves AKT/GSK or NFκB-mediated expression of Snail and promotes cell invasion and migration in various cancers, such as breast, renal, and colon cancers [10, 11]*.* The loss of E-cadherin, an adherens junction cell surface protein expressed in epithelial cells is the principal characteristic of EMT [12]. The Snail and Slug signaling cascades are among those that may be involved in EMT in cancer cells. Snail and Slug are key transcription factors that can down regulate the expression of E-cadherin. They do this by binding to E-boxes in the E-cadherin promoter, subsequently increasing MMP-9 expression to promote cell invasion [13]. However, few studies have investigated the suppression of molecular events and EMT responsible for EMT inhibition in anticancer treatment.

The Wnt/β-catenin signaling pathway contributes to cell fate decisions as well as the normal cellular response during cancer cell development [14]. Researchers have suggested that dysregulated or uncontrolled triggering of this signaling pathway promotes tumor progression and metastasis in patients with breast cancer [15]. Other attributes of the Wnt extracellular signaling pathways manage tissue architecture, proliferation, embryonic axis formation, and cell migration [16] and can be broadly classified into noncanonical and canonical pathways. Canonical pathways are activated when the relevant Wnt ligands bind to the LRP-5/6 coreceptors and Frizzled transmembrane domain receptor [17], whereas non-canonical pathways are β-catenin-independent and need Ror2/Ryk coreceptors rather than LRP-5/6 coreceptors. β-Catenin is usually aberrantly activated in breast cancer tissues. Therefore, Wnt/β-catenin pathway inhibition has the potential to reduce breast cell invasion as well as that of their EMT.

Coenzyme Q_0_ (CoQ_0)_ also known as ubiquinone 0 and 2,3 dimethoxy-5-methyl-1,4 benzoquinone) and a member of the mitochondrial respiratory chain is a redox-active ubiquinone compound commonly present in the mitochondrion. It possesses strong antioxidant activity and prevents the mitochondrial permeability transition pore [18] from being opened calcium-dependently. CoQ_0_ has demonstrated activity against the proliferation of numerous cancer cell lines (e.g., HepG2, A549, and SW480) [19, 20]. Although it exhibits cytotoxic anticancer activities, it was also demonstrated to stimulate insulin secretion in pancreatic islets [21]. We described its anti-inflammatory and anti-angiogenic properties in vivo and in vitro in our previous study [22]. Remarkably, administering CoQ_0_ mixtures prevents oxidative damage in rodent spleen, blood, kidney, heart, and liver [23]. Our previous study on CoQ_0_ found that it significantly inhibits melanoma cell growth and tumor formation by inducing apoptosis and cell-cycle arrest [24]. Additionally, it effectively promoted apoptosis by increasing ROS in MCF-7 cells that were irradiated using ultraviolet B [22]. Despite CoQ_0_’s anticancer attributes, its inhibitory effect on breast cancer metastasis and EMT and the molecular mechanism that gives it its therapeutic efficacy are unclear.

To ascertain CoQ_0_’s capabilities at inhibiting metastasis, EMT, and their associated changes, we designed a validated EMT and metastasis model for human TNBC (MDA-MB-231). Metastasis and EMT control levels and the principal molecular biomarkers involved were analyzed to ascertain the anti-EMT and antimetastatic attributes mediated by CoQ_0_. In addition, we sought to clarify the fundamental mechanism of TNBC cells.

## Methods and materials

### Reagent and antibodies

CoQ_0_ was from Sigma-Aldrich (St. Louis, MO, United States), as was the 3-(4,5-dimethylthiazol-2-yl)-2,5-diphenyltetrazolium bromide reagent for the MTT assay. GIBCO BRL/Invitrogen (Carlsbad, CA, United States) supplied _L_-glutamine, penicillin/streptomycin/neomycin, and Dulbecco’s modified Eagle’s medium (DMEM). Antibodies against anti-NFκB (p65), phos-IKK, Cyclin D1, CDK4, PARP, β-catenin, p-AKT, p-PI3K, PI3K, H3 antibodies, and IKK were from Cell Signaling Technology Inc. (Danvers, MA, United States), and those against MMP-9, vascular endothelial growth factor (VEGF), β-actin, c-Myc, Bax, Bcl-2, p21, p27, p53, caspase-3, cytochrome C, and I-κB were from Santa Cruz Biotechnology, Inc. (Heidelberg, Germany). The remaining chemicals were of HPLC grade and were from either Sigma-Aldrich or Merck (Darmstadt, Germany).

### Generation of breast cancer cell lines

The tumorigenic triple-negative (MDA-MB-231, MDA-MB-231-Brain, MDA-MB-231-Brain-erb2, MDA-MB-231-Bone, MDA-MB-231-Bone-erb2, BT549, Hs578T), non-tumorigenic MCF-10A, and estrogen receptor-positive (BT474 and MCF-7) cell lines were from ATCC (Manassas, VA, United States). Cell lines underwent culturing at 37 °C in DMEM supplemented with 10% fetal bovine serum (FBS), 2 mM L-glutamine, and 1% streptomycin. Furthermore, MCF-10A cells were grown at 37 °C in DMEM/F12 supplemented with 2 mM glutamine, 5% horse serum, 0.5 μg/mL hydrocortisone, 20 ng/mL human epidermal growth factor, 10 μg/mL insulin, and 1% streptomycin.

### MTT assay

MTT colorimetric assays were employed to ascertain cell viability [25]. Cells were grown in 12-well plates to a confluence. Subsequently, they underwent 24-h CoQ_0_ incubation (2.5–20 μM). Following the MTT assay, an ELISA microplate reader (Bio-Tek Instruments Inc., Winooski, VT, United States) was utilized to ascertain the absorbance at 570 nm. The proportion of viable cells relative to vehicle-treated control cells (designated as 100%) was the basis for evaluating CoQ_0_’s effect on cell viability.

### In vitro wound-healing assay

The effects of CoQ_0_ on cell migration were evaluated using in vitro wound-healing assay as follows: the cells were cultured (density: 1 × 10^4^), and the standard protocol of the in vitro healing assay was followed [26]. Subsequently, the cells underwent 24-h incubation with varying CoQ_0_ concentrations (0.5–2 μM) in 1% FBS medium followed by phosphate-buffer saline (PBS) wash (3 times). They were subsequently fixed using 100% methanol. Finally, they were stained using Giemsa staining solution (Merck, Darmstadt). Migrated cells were monitored, and phase-contrast microscopy at 200× was used to photograph them. Image-Pro Plus (Media Cybernetics, Inc., Rockville, MD, United States) was used to compute the wounded area’s closure.

### Cell invasion assay

Cell invasion assay was conducted using BD Matrigel invasion chambers (Bedford, United States), as described by Debbie and Brooks [27]. We used 10 μL of Matrigel (25 mg/50 mL) to coat 8-μm polycarbonate membrane filters. Then, we seeded the cells (density: 1 × 10^5^) onto the Matrigel-treated filter in 200 μL of CoQ_0_ (0.5–2 μM) devoid of serum. Cell migration underwent 24-h observation at 37 °C. After being incubated for 24 h, the cells that did not migrate were removed from atop the membrane by using a cotton swab. On the other side of the membrane, the migrated cells were fixed for 15 min in ice-cold methanol (75%) and were washed in PBS three times. Subsequently, Giemsa stain was used to fix the cells; they were subsequently destained using PBS. Images were viewed and captured through 200× light microscopy; we quantified invading cells by using manual counting. All experiments were repeated three times.

### Protein isolation and Western blotting

We seeded the cells (density: 4 × 10^6^ cells/dish) into a 6-cm dish that had undergone 24-h CoQ_0_ (2.5–10 μM) pre-treatment. Next, they underwent trypsinization and 3 times rinsing with ice-cold PBS. Total cytoplasmic and nuclear extracts were isolated in accordance with the suggested protocol (Pierce Biotechnology, Waltham, MA, United States). The protein concentration was ascertained using Bradford reagent, with bovine serum albumin (BSA) as standard. The lysate was separated through 12% SDS-PAGE. PVDF membranes were immunoblotted using specific primary antibodies and their corresponding horseradish peroxidase–conjugated secondary antibodies. An enhanced chemiluminescence substrate (Pierce Biotechnology, Waltham) was utilized to develop the blot, and a densitometric graph displaying band intensities was generated using AlphaEaseFC (Miami, FL, United States).

### RNA extraction and RT-PCR

After cells had undergone 24-h pre-treatment with various CoQ_0_ concentrations (0.5–2 μM), they were harvested. Subsequently, TRIzol reagent (Invitrogen, Carlsbad) was employed to extract total RNA. Then, Bio-Rad iCycler PCR instrument (Bio-Rad, Hercules, CA, United States) and SuperScript-III One Step RT-PCR platinum *taq* kit (Invitrogen, Carlsbad) were used for RT-PCR of 1 μg of total RNA in accordance with the procedure [28]. The PCR product was analyzed in agarose gel (1%). The primers used were as follows: MMP-2 F: 5′ATGACAGCTGCACCACTGAG-3′, R-5′-ATTTGTTGCCCAGGAAAGTG -3′; MMP-9 F-5′- TTGACAGCGACAAGAAGTGG-3′, R-5′-GCCATTCACGTCGTCCTTAT-3′; uPA F-5′-TGCGTCCTGGTCGTGAGCGA-3′, R-5′-CAAGCGTGTCAGCGCTGTAG-3′; uPAR F-5′CATGCAGTGTAAGACCCAACGGGGA-3′, R-5′-AATAGGTGACAGCCCGGCCAGAGT3′; E-cadherin: F-5′-TGGGTTATTCCTCCCATCAG-3′, R-5′TTTGTCAGGGAGCTCAGGAT-3′; Vimentin: F- 5′CTCTTCCAAACTTTTCCTCC 3′, R-5′AGTTTCGTTGATAACCTGTC 3′; Snail: F-5′CGAAAGGCCTTCAACTGCAAAT 3′, R-5′ACTGGTACTTCTTGACATCTG 3′; Slug: F- 5′CGCCTCCAAAAAGCCAAAC 3′, R-5′CGGTAGTCCACACAGTGATG 3′; β-catenin: F-5′-AAGGAAGCTTCCAGACATGC 3′, R-5′AGCTTGCTCTCTTGATTGCC 3′; 18S F-5′GTCTGTGATGCCCTTAGATG 3′, R-5′AGCTTATGACCCGCACTTAC 3′.

### Mammosphere formation assay

We performed a mammosphere formation assay as described elsewhere with several minor modifications [29]. For mammosphere culture from MDA-MB-231 cells, we suspended cells at 1 × 10^3^ cells/mL and seeded them onto ultralow attachment plates (Corning Inc., Corning, NY, United States) in MammoCult basal medium (STEMCELL Technologies, United States) supplemented with 0.2% heparin (STEMCELL Technologies, United States), 500 μg/mL cortisone (STEMCELL Technologies, United States), and 10 μL/mL mammo supplant serum (STEMCELL Technologies, United States) in an incubator supplemented with 5% CO_2_. The cells underwent dimethyl sulfoxide (DMSO) (0.1%) or CoQ_0_ (0.5–2 μM) treatment and were then incubated for 1 week during which time the plate was kept stationary and the media were replenished. On day 7, the mammospheres were collected through gentle centrifugation and dissociated into single-cell suspensions; the cells then underwent repeated vehicle control (0.1% DMSO) or CoQ_0_ (0.5–2 μM) treatment. The cell suspensions underwent three passages through a syringe with 26-G needles. This procedure resulted in single-cell suspensions being formed. We then adjusted cell concentration to 500 cells/mL and plated the 2-mL single-cell suspensions on a 35-mm ultralow attachment plate (Corning Inc., Corning) in triplicate. For serial passage (secondary sphere formation) and differentiation experiments, this study employed early progenitor cells as well as sphere forming, breast stem–enhanced single cells. On day 14, a graticule-enabled microscope was employed to determine the number of mammospheres of diameter > 50 μm as well as the number of colony-forming units. Then, the cells were trypsinized, and the cell numbers were counted. Two independent observers not exposed to the treatment were asked to count the sphere numbers manually. All experiments were conducted three times.

### Colony formation assay

MDA-MB-231 cells (density: 5 × 10^5^ cells/60-mm dish) underwent 24-h CoQ_0_ treatment (concentrations: 0–7.5 μM). We then trypsinized the cells and replated them (density: 3 × 10^4^ cells/35-mm dish) in triplicate. They were subsequently subjected to 7-d RPMI 1640 incubation. Afterward, they were fixed at room temperature for 10 min using 10% neutral buffered formalin and stained using 20% Giemsa stain (Merck, Darmstadt). We subsequently assayed the cells for ability to form colonies and proliferation rate. The number of colonies of size > 1 mm was determined with light microscopy (40×). Colony number was ascertained based on a colony formation percentage of 100% in the absence of CoQ_0_.

### Gelatin zymography assay

The zymography protease assay was used to quantify the activity of MMP-2 and 9 in the medium used to grow the MCF-10A cells. The MCF-10A cells (1 × 10^6^ cells/well) were seeded into 6-well culture dishes and grown in medium with 10% FBS to a nearly confluent monolayer. The cells were re-suspended in 1% FBS medium, and then incubated with TGF-β/TNF-α (10 ng/mL) and CoQ_0_ (2 μM) for 24 h. After treatment the remains procedures were followed according to the previously mentioned work [30]. The changes in expression of MMP-2 and -9 were quantified by Matrix Inspector 2.1 software (AlphaEase, Genetic Technology, Inc., Miami, FL, USA).

### Immunofluorescence staining

Cells (density: 1 × 10^4^ cells/well) underwent culturing; this culturing was performed in an eight-well glass Tek chamber. Subsequently, CoQ_0_ (0.5–2 μM) was used to pre-treat them for the recommended duration. Next, they underwent 15-min fixing in paraformaldehyde (2%), 10-min Triton (0.1%) permeabilization, washing, and FBS (10%) blocking in PBS. Accordingly, the cells underwent 2-h primary antibody (anti-p65, anti-Vimentin, anti-F-actin, anti-β-catenin, and anti-E-cadherin) incubation in 1.5% FBS. Subsequently, they were incubated for 1 h using FITC-conjugated secondary antibody in BSA (6%). We then stained them for 5 min using 1 μg/mL DAPI. The cells were subsequently subjected to secondary antibody incubation and DAPI staining, washed thoroughly using PBS, and then visualized through fluorescent confocal microscopy.

### Luciferase reporter assay

The transcriptional activity of NFκB, E-cadherin, and β-catenin was ascertained through dual-luciferase reporter assays (Promega, Fitchberg, WI, United States). We grew the cells to 70–80% confluence for 5 min in a 24-well plate by using serum-free DMEM devoid of antibiotics. They were subsequently transfected with NFκB, E-cadherin, or β-catenin plasmids with β-galactosidase or a pcDNA vector by using Lipofectamine 2000 (Invitrogen, Carlsbad). Afterward, they underwent 4-h CoQ_0_ (0.5–2 μM) treatment. Subsequently, they were lysed, following which their luciferase activity was ascertained through luminometry (Bio-Tek, Winooski) and then standardized to their β-galactosidase activity in the cell lysates. In addition, we quantified the intensity of relative fluorescence by using a luminance ELISA reader.

### Immunoprecipitation

Protein A-sepharose beads were used to preclear 1 mg of the protein sample for 1 h, which subsequently underwent 24-h incubation using 2 mg of anti-E-cadherin antibody. Radioimmunoprecipitation assay (RIPA) buffer was employed to wash the immunoprecipitated complexes. Subsequently, the complexes were denatured using SDS sample buffer. Centrifugation (14,000 rpm) was employed to clarify the cell lysate for 15 min. It was then separated through SDS-PAGE and conveyed to PVDF membranes. Skim milk (5%) was used to block the membrane for 30 min. Then, it was immunoblotted using specific primary antibodies and the corresponding HRP-conjugated secondary antibodies for 1 h and subsequently visualized with the aid of an electroluminescence reagent (Millipore, Burlington, MA, United States).

### Transient transfection of siRNA targeting β-catenin

Lipofectamine RNAiMAX (Invitrogen, Carlsbad) was used to transfect the cells with β-catenin siRNA. To facilitate transfection, the cells were incubated in 10% FBS containing DMEM; subsequently, they were plated to 60% confluence on a 6-well plate during transfection. The following day, we replaced the culture medium with 500 μL of Opti-MEM, which we then subjected to transfection using 5 μL of RNAiMAX.

We mixed 250 μL of Opti-MEM and 5 μL of RNAiMAX and subjected the mixture to incubation for 5 min at room temperature. In another tube, 100 pM siRNA in 1 mL of Opti-MEM and 250 μL of Opti-MEM were combined. Subsequently, we added siRNA solution to the diluted RNAiMAX reagent, and the prepared 500-μL siRNA/RNAiMAX mixtures underwent incubation at room temperature for 25 min to facilitate the formation of the complex.

Afterward, the cells and solution were combined. They together represented a final transfection volume of 1 mL. After 6-h incubation, 2 mL of standard growth medium was used to replace the transfection medium, which subsequently underwent culturing at 37 °C. Afterward, the cells underwent 24-h CoQ_0_ (1 μM) incubation. To ascertain cellular protein level, Western blotting was employed.

### Analysis of F-actin distribution and cell morphology

The cells (1 × 10^4^ cells/8-chamber slide) were cultured in DMEM or high glucose that contained FBS (10%). Twenty-four hours later, the cells underwent 1-h CoQ_0_ (2 μM) pre-treatment. Subsequently, they were activated using TGF-β/TNF-α (10 ng/mL) for 24 h. We then fixed them in paraformaldehyde (3.7%), blocked them in BSA (3%), and stained the F-actin using TRITC-conjugated phalloidin in order to monitor the actin cytoskeleton; to image the nuclei, DAPI (1 μg/mL) was added. Fluorescence microscopy (magnification: 100×; Nikon, Tokyo, Japan) was employed to capture images.

### Cell-cycle analysis

Cellular DNA content was assessed through flow cytometry using propidium iodide (PI)–labeled cells. DMEM medium with thymidine (3 mM) was used to block AGS cells for 16 h. PBS was subsequently employed to wash cell-cycle synchronized cells, and these cells were reprogrammed so as to enter the G_1_ phase for 24 h with fresh DMEM medium added that included CoQ_0_ (5–15 μM). Afterward, cellular trypsinization was executed, and the cells were subsequently fixed at − 20 °C overnight in 3 mL of ice-cold ethanol (70%). Their pellets were gathered through centrifugation and resuspended in 0.5 mL of PI staining buffer (1% Triton x-100, 0.5 mg/mL RNase A, 4 μg/mL PI in PBS). They were then incubated at room temperature for 30 min. FACScan cytometry (BD Biosciences, San Jose, CA, United States) adapted with a 488-nm single Ar ion laser was adopted to ascertain cell-cycle progression. ModFit (Verity Software House, Topsham, ME, United States) was utilized to analyze the cell cycles.

### TUNEL assay

Apoptotic cell death was examined by applying the TUNEL method by using TdT-dUTP-fluorescein in situ cell detection tools (Roche, Mannheim, Germany) as expounded elsewhere [31]. Optical microscopy was used to view the slides.

### Assay for cell apoptosis rate

We conducted double staining for PI and Annexin V-FITC to evaluate MDA-MB-231 cells’ apoptotic rate. In short, cells underwent 24-h CoQ_0_ (5–15 μM) incubation, trypsinization, 2× PBS washing, and 5-min 800-rpm centrifugation. Next, we suspended the cells (1 × 10^6^ cells/10-cm dish) in 100-μL binding buffer and double stained them using a PI Apoptosis Detection kit (BioVison, Mountain View, CA, United States) and Annexin-V-FITC. Subsequently, the resultant red (PI) and green (FITC) fluorescence for the samples was quantitatively analyzed through FACSCaliber flow cytometry (Becton Dickinson, Franklin Lakes, NJ, United States). Finally, data were processed on CellQuest (BD Biosciences, San Jose).

### Measurement of ROS level in cells

The cells (density: 4 × 10^5^ cells/well) were grown in a 12-well plate. They underwent 5 min–1 h of CoQ_0_ (5–15 μM) pre-treatment. Following, DCFH_2−_ (10 μM) and the culture were combined; they were subjected to incubation for 30 min at 37 °C. Warm PBS was used to wash the cells, and generation of ROS was ascertained from intracellular DCF production that was the result of DCFH_2_ oxidation [32]. A fluorescence microscope (Olympus, Shinjuku, Tokyo) was employed (magnification: 200×) to determine the level of DCF fluorescence.

### Xenograft animal model

China Medical University’s Institutional Animal Care and Treatment Committee approved all protocols involving animals and their welfare. Briefly, 5- to 7-week-old athymic nude mice (female; BALB/*c-nu*), acquired from the National Laboratory Animal Center (Taipei, Taiwan), were confined in a sterile environment with a 12–12 h light–dark cycle. They were fed rodent chow (Oriental Yeast Co., Tokyo) and provided unlimited access to water.

The mice were subcutaneously engrafted in the right-hind flank with 1 × 10^6^ MDA-MB-231 cells. The mice were separated into two groups of five. For this experiment, cells passaged less than 20 times were used. The treatment-group mice were administered C0Q_0_ (0.75 mg/kg b.w.) Intraperitoneal (IP) injection (three times/week) was performed for 12 weeks; the control group received only the vehicle (PBS). The tumors were measured on a weekly basis, and tumor volume was ascertained as width^2^ × length × 0.5 (mm). The mice were sacrificed on the 12th week; this was followed by tumor removal and weighing. A veterinary pathologist examined the excised organs, including the liver, lungs, and kidneys.

### In vivo anti-metastasis by bioluminescence imaging

The animals that underwent CoQ_0_ (1.5 or 2 mg/kg) and MDA-MB-231-luciferase cell (1 × 10^6^ cells/well) treatment were injected intravenously. Afterward, they were sedated and then IP injected with luciferin. An IVIS 200 system was employed to image them, with the images representing the luminescence (photons/s) emitted from the animal.

### Immunohistochemistry analyses

Paraformaldehyde (4%) was used to fix the biopsied tumor tissues. This, in addition to segmentation and staining, were performed to facilitate observation under light microscopy. For Western blotting, the tissue samples were mixed in RIPA buffer that contained phosphatase inhibitor cocktail (1%; Sigma-Aldrich, St. Louis) and protease inhibitor cocktail (1%). SDS-PAGE gel was the site of sample separation. Subsequently, the samples were relocated onto PVDF membranes. Corresponding primary and biotinylated secondary antibodies (Zymed Laboratories, South San Francisco, CA, United States) were added to the PVDF membranes and incubated. Afterward, the membranes underwent avidin-biotin complex reagent incubation. Finally, they were stained using 3,3′-diaminobenzidine in accordance with the manufacturer’s procedures (Histostain-Plus Kit; Zymed, South San Francisco).

### Statistical analysis

The experiment was conducted in triplicate; the values were represented as mean ± standard error. Significance was ascertained using Dunnett’s test for pair-wise comparison.

## Results

### CoQ_0_ inhibits colony formation and cell viability of breast cancer cells

**Fig. 1:**
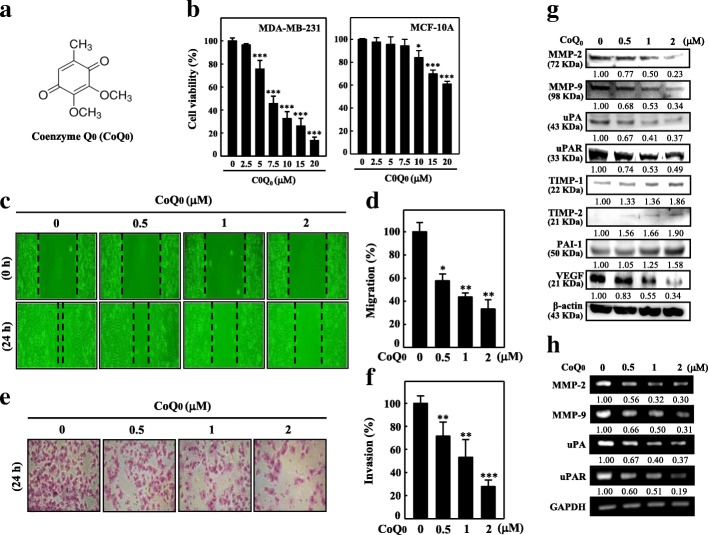
CoQ_0_ inhibits proliferation, cell migration and invasion in human breast cancer cells. **a** Structure of CoQ_0_. **b** Human tumorigenic breast cancer cell lines TNBC, MDA-MB-231, and non-tumorigenic line MCF-10A were treated with CoQ_0_ (2.5–20 μM) or vehicle control (0.1% DMSO) for 24 h. MTT colorimetric assay was used to determine cell viability. **c**-**f** CoQ_0_ inhibits migration and invasion of MDA-MB-231 cells. Cells MDA-MB-231 were treated with corresponding concentration of CoQ_0_ for 24 h. **c**-**d** Cells were scratched, and migration was observed by an optical microscope (200 × magnification). The area closure was calculated by commercially available software. **e**-**f** Invasiveness was determined by counting per sample three microscopic fields. The inhibitory percentage of invading cells was quantified and expressed with untreated cells (control) representing 100%. **g**-**h** CoQ_0_ modulates metastatic-related proteins of MDA-MB-231 cells. Cells were treated with CoQ_0_ (0.5, 1, or 2 μM) for 24 or 4 h. CoQ_0_ mediated downregulation of MMP-2, MMP-9, uPA, uPAR, and VEGF expression, and up-regulation of their endogenous inhibitors TIMP-1, TIMP-2, and PAI-1, as measured by Western blot (24 h) (**g**) or RT-PCR (4 h) (**h**) analyses. The results are presented as the mean ± SD of three independent assays **p < 0.05, ***p < 0.001 significant compared to control cells

The cytotoxic role of CoQ_0_ (Fig. 1a) in the non-tumorigenic MCF-10A and MDA-MB-231 cell lines was investigated. When the cells underwent 24-h treatment with increasing concentrations of CoQ_0_ (i.e., 2.5, 5, 7.5, 10, 15, and 20 μM), growth inhibition was noted in MDA-MB-231, indicating cytotoxicity (Fig. 1b); by contrast, in MCF-10A cells, minimal cytotoxicity was exhibited. These results indicated that CoQ_0_ was more potent against TNBCs than against non-tumorigenic cells (Fig. 1b).

Tumor local invasion and migration comprise the first stage in cancer cell metastatic cascade, resulting in the production of typically fatal distant metastasis. The influence of CoQ_0_ (0.5–2 μM) on MDA-MB-231 cell migration was investigated through an in vitro wound closure assay followed by a scratch assay. A significant dose-dependent decrease in cell migration was noted for CoQ_0_ treatment (Fig. 1c-d). Then, the ability of CoQ_0_ to hinder invasion of MDA-MB-231 cells was determined using BD Matrigel chamber assay. CoQ_0_ (0.5–2 μM, 24 h) significantly and dose-dependently decreased the invasion of MDA-MB-231 (Fig. 1e-f). The foregoing results suggest that treatment with CoQ_0_ has antimigratory and anti-invasive impacts on MDA-MB-231 cells.

### CoQ_0_ mediates downregulation of MMPs, uPA, and VEGF and upregulation of TIMPs and PAIs in MDA-MB-231 cells

uPA and MMPs, which play key roles in basement membrane degradation, are vital actors in invasion and migration. To determine the impact of CoQ_0_ on the levels of uPA and MMPs, MDA-MB-231 cells underwent 24-h CoQ_0_ (0.5–2 μM) treatment. A substantial reduction in the protein expression of MMP-2, MMP-9, uPAR, and uPA was detected (Fig. 1g). Western blotting revealed that VEGF expression was downregulated following CoQ_0_ treatment (Fig. 1g). MMP and uPA physiological activity is associated with PAIs and TIMPs irrespective of their particular endogenous inhibitors. Accordingly, we studied TIMP and PAI expression in the MDA-MB-231 cells that underwent 24-h CoQ_0_ (0.5–2 μM) treatment. Substantial upregulation in the expression of PAI-1, TIMP-1, and TIMP-2 was recorded after CoQ_0_ treatment (Fig. 1g). Furthermore, CoQ_0_ in MDA-MB-231 inhibited the mRNA gene expression of uPA, uPAR, MMP-9, and MMP-2 for the tested concentrations (Fig. 1h). The results suggest that MMP-9, which is a crucial factor for metastasis, was inhibited by CoQ_0_ treatment.

### COQ_0_ attenuates mammosphere formation in MDA-MB-231 cells

**Fig. 2:**
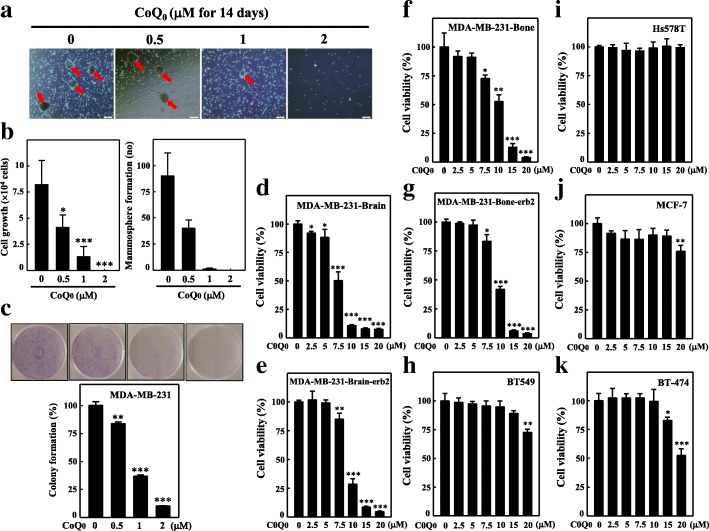
CoQ_0_ attenuates mammosphere formation and colony formation in triple-negative breast cancer MDA-MB-231 cells. **a**-**b** CoQ_0_ attenuates mammosphere formation. Cells were treated with CoQ_0_ (0.5–2 μM) or vehicle control (0.1% DMSO) for 14 days. **b** The cell growth and mammosphere formation were analyzed by MTT assay and mammosphere formation assay, respectively. **c** CoQ_0_ inhibits colony formation ability. Cells were assayed for their ability to proliferate and form colonies in the presence of CoQ_0_ (0.5–2 μM) and were incubated 5 days. The percentage of colony formation was calculated by defining the number of colonies in the absence of CoQ_0_ as 100%. **d**-**j** Human breast cancer cell lines MDA-MB-231-Brain (**d**), MDA-MB-231-Brain-erb2 (**e**), MDA-MB-231-Bone (**f**), MDA-MB-231-Bone-erb2 (**g**), BT549 (**h**), Hs578T (**i**), MCF-7 (**j**), and BT474 (**k**) were treated with CoQ_0_ (2.5–20 μM) or vehicle control (0.1% DMSO) for 24 h. The cell viability was determined by MTT colorimetric assay. The results are presented as the mean ± SD of three independent assays. **p < 0.05, ***p < 0.001 significant compared to control cells

A previous research depicted the development of mammospheres, which are spherical clusters of nonadherent mammary stem/progenitor cells [33]. To investigate whether CoQ_0_ affects tumor cell mammosphere formation, we exposed cells to various concentrations of CoQ_0_ for 14 d. Intriguingly, our results indicate that CoQ_0_ inhibited nonadherent spherical breast cancer clusters in vitro so that the cells became noncompetent in generating secondary spheres and differentiating along more than one lineage (Fig. 2a-b). As indicated in Fig. 2a, reduction in the size and formation of such spheres was observed for CoQ_0_ (0.5, 1, and 2 μM) treatment. Subsequently, we determined the differentiation and proliferation capacity of these cells in the presence of CoQ_0_. A significant reduction in cell growth of MDA-MB-231 was noted (Fig. 2b). The collective outcome of the aforementioned results suggests that CoQ_0_ exhibits a strong anti-mammosphere forming capability.

### CoQ_0_ attenuates colony formation

Subsequently, we determined whether CoQ_0_ could affect soft agar–cultured MDA-MB-231 cells anchorage-independent growth, a property that tumor cells demonstrate with in vivo tumorigenesis. Dose-dependent inhibition (5 d) of the growth of anchorage-independent MDA-MB-231 cells was noted (Fig. 2c). Colony number inhibition increased with a reduction in colony size. The decreased colony formation ability with CoQ_0_ indicates reduced MDA-MB-231-cell tumorigenic ability. These results reveal that CoQ_0_ effectively hinders MDA-MB-231-cell survival and growth.

### CoQ_0_ inhibits breast cancer cells viability

CoQ_0_ and its cytotoxic effects on the proliferation of TNBC MDA-MB-231-Brain, MDA-MB-231-Brain-erb2, MDA-MB-231-Bone, MDA-MB-231-Bone-erb2, BT549, Hs578T, BT474, and estrogen receptor-positive MCF-7 were explored. Cells underwent 24-h treatment with 0–20 μM concentrations of CoQ_0_. Significant cytotoxic effects were observed in the MDA-MB-231-Bone, MDA-MB-231-Bone-erb2, MDA-MB-231-Brain, and MDA-MB-231-Brain-erb2 cell lines dose dependently (Fig. 2d-g), whereas CoQ_0_ showed no to minimal effect against BT549, Hs578T, MCF-7, and BT474 (Fig. 2h-k). These results indicated that CoQ_0_ was more potent against the brain and bone metastatic variants of TNBC cells than against other tumorigenic cells.

### CoQ_0_ attenuates NFκB activation by suppressing I-κBα degradation in MDA-MB-231 cells

**Fig. 3:**
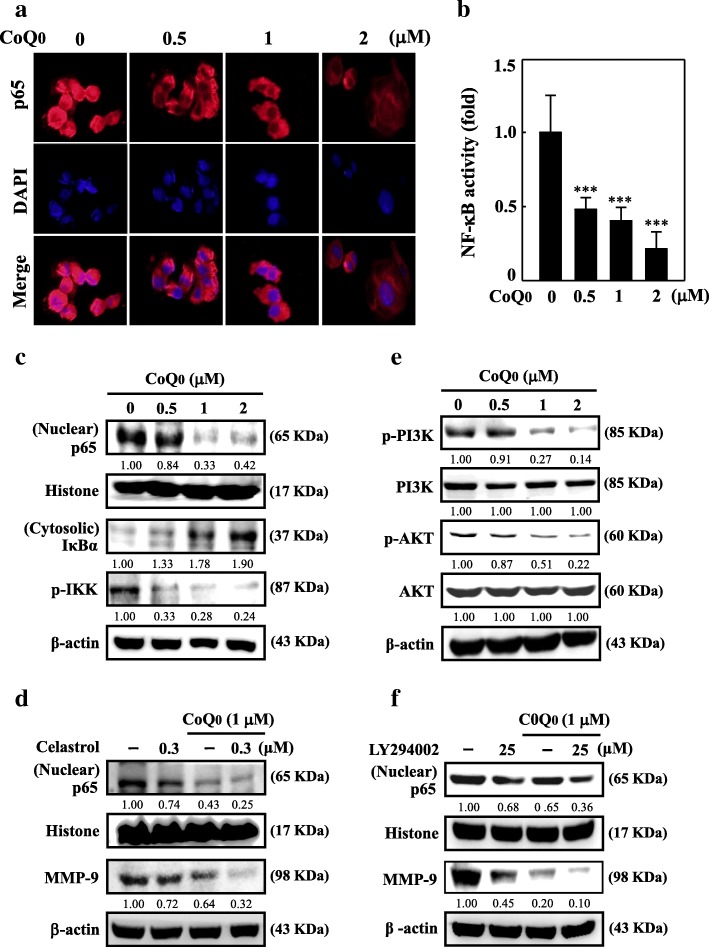
CoQ_0_ inhibits metastasis through the downregulation of PI3K/AKT/NFκB signaling pathways in MDA-MB-231 cells. **a** Cells were grown in chamber slides and exposed to CoQ_0_ *(*0.5–2 μM) for 2 h, fixed and permeabilized. Cells were incubated with anti-p65 antibody followed by FITC-labeled secondary antibody. The subcellular localization of p65 was visualized using a confocal microscope of 40 × magnification. **b** NFκB activity was evaluated via luciferase reporter gene assay. Cells were transfected with the luciferase reporters and co-transfected with either NFκB or empty vector. Then, they were treated with CoQ_0_ (0.5–2 μM) for 4 h, and luciferase activity was determined and normalized with β-gal activity, shown as relative luciferase activity. **c** CoQ_0_ induces nuclear p65 and inhibits cytosolic I-κB and p-IKK degradation. Cells were treated with *CoQ*_0_ *(*0.5–2 μM for 2 h). Cytoplasmic and nuclear extracts were prepared and Western blot analyses were performed. β-actin or histone H3 was used as an internal control for cytoplasmic and nuclear extracts, respectively. **d** CoQ_0_ inhibits MMP-9 expression through the downregulation of NFκB signaling pathways. The nuclear p65 (for 4 h) and MMP-9 expression for 24 h was inhibited following the treatment with CoQ_0_ *(*1 μM) in the presence or absence of NFκB inhibitor celastrol (0.3 μM), as shown by Western blot. For internal control β-actin was used. **e** Phosphorylated PI3K (p-PI3K) and AKT (p-AKT) levels were evaluated by immunoblot analysis. Cells were treated with *CoQ*_0_ *(*0.5–2 μM) for 24 h. β-actin, total PI3K and AKT levels were considered as an internal control. **f** CoQ_0_ inhibits NFκB/MMP-9 expression through the downregulation of PI3K/AKT signaling pathways. The nuclear p65 (for 4 h) and MMP-9 expression (for 24 h) was inhibited following treatment with CoQ_0_ *(*1 μM) in the presence of absence of PI3K/AKT inhibitor LY294002 (25 μM), as shown by Western blot. β-actin was used as an internal control. The results are presented as the mean ± SD of three independent assays. **p < 0.05, ***p < 0.001 significant compared to control cells

The NFκB transcription factor family regulates myriad genes involved in MMP, VEGF, or uPA expression. Therefore, the effect of CoQ_0_ on NFκB signaling and its regulatory proteins in MDA-MB-231 cell were studied. Immunofluorescence assay shows that CoQ_0_ treatment diminished nuclear p65 protein dose dependently (Fig. 3a). NFκB activity, which was ascertained through luciferase reporter assays, was high in the control group but decreased dose dependently in cells treated using CoQ_0_ (Fig. 3b).

Next, we determined whether the inhibition of NFκB by CoQ_0_ is related to I-κBα protein degradation, which regulates NFκB stability. CoQ_0_ treatment attenuated I-κBα degradation, which in turn hindered NFκB (p65) activation (Fig. 3c). To determine whether I-κBα degradation suppression was the result of IKKα phosphorylation inhibition, we used Western blotting to explore IKKα phosphorylation. CoQ_0_ pre-treatment suppressed IKKα phosphorylation dose dependently (Fig. 3c). These results indicate that CoQ_0_ suppressed nuclear activation of NFκB by inhibiting I-κBα degradation.

### CoQ_0_ inhibits MMP-9 through the inhibition of PI3K/AKT/NFκB pathways in MDA-MB-231

NFκB has a major role in managing the MMP expression in numerous cancer cell lines. To more profoundly comprehend the inhibitory mechanisms of CoQ_0_ on the transcriptional regulation of MMP-9, we investigated NFκB and MMP-9 through Western blotting. The cells underwent celastrol (an NFκB inhibitor) pre-treatment and then 24-h CoQ_0_ treatment. The results revealed that CoQ_0_ lowered p65 and MMP-9 protein expression. However, inhibition was more pronounced in the case of celastrol pre-treatment (Fig. 3d).

Based on the findings that CoQ_0_ inhibited NFκB and MMP-9 expression and because PI3K/AKT is the major pathway involved in NFκB/MMP-9 activation, we hypothesized that CoQ_0_ regulates the PI3K/AKT pathway. We evaluated this hypothesis by appraising the impact of CoQ_0_ on PI3K and AKT phosphorylation. As shown in Fig. 3e, CoQ_0_ substantially decreased p-PI3K and p-AKT expression dose dependently. Subsequently, we investigated the impact of LY294002, a PI3K inhibitor, on nuclear p65 and MMP-9 expression in the absence or presence of CoQ_0_. Lower nuclear p65 and MMP-9 expression was recorded in the presence of LY294002 (Fig. 3f). These results suggest that CoQ_0_ suppressed MMP-9 by inhibiting the PI3K/AKT/NFκB pathways of MDA-MB-231 cells.

### CoQ_0_ suppresses EMT by restoring E-cadherin pathways in MDA-MB-231

**Fig. 4:**
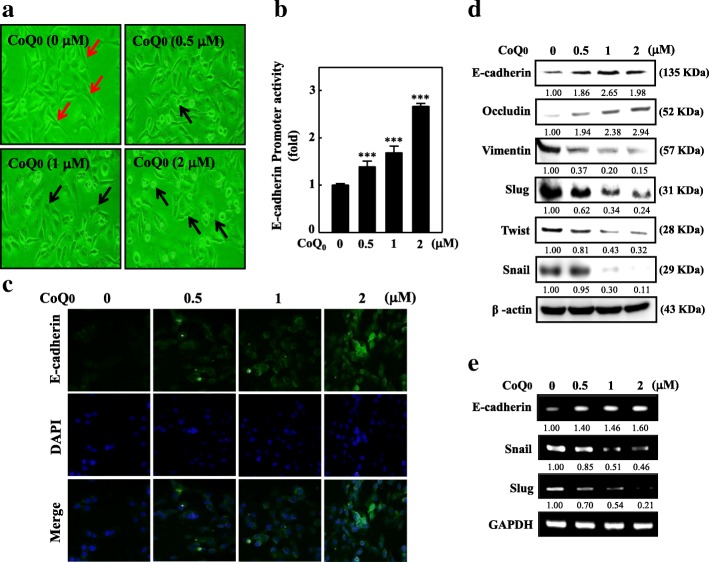
CoQ_0_ inhibits EMT through up-regulation of E-cadherin signaling pathways in MDA-MB-231 cells. **a**-**d** Cells were treated with CoQ_0_ (0.5–2 μM) for 24 h. **a** Morphological changes were examined by phase-contrast microscope (200× magnification). **b** Transcriptional activity of E-cadherin was monitored by luciferase reporter assay. **c** Immunofluorescence analysis for E-cadherin protein expression. **d** CoQ_0_-induced modulation of epithelial (E-cadherin and Occludin) and mesenchymal marker proteins (Vimentin, Slug, Twist, and Snail) were monitored using Western blot analyses. **e** mRNA expression of E-cadherin, Vimentin, Slug, and Snail after 6 h treatment with CoQ_0_ (0.5–2 μM) was measured by RT-PCR analyses. As internal control GAPDH was used. The results are presented as the mean ± SD of three independent assays. **p < 0.05, ***p < 0.001 significant compared to control cells

Incubating cells by using varying CoQ_0_ concentrations (0.5–2 μM) for 24 h resulted in abrupt morphological changes compared with the control group (Fig. 4a). E-cadherin, a protein that has a role in regulating EMT, was assayed in MDA-MB-231 cells. A rise in E-cadherin expression due to CoQ_0_ may be because E-cadherin transcriptional activity was activated. Thus, we measured E-cadherin promoter activity on the basis of luciferase activity: the data are provided in Fig. 4b. We transfected an E-cadherin promoter construct in a pcDNA vector into MDA-MB-231 cells; we then ascertained cell luciferase activity. The findings indicated that luciferase activity from the E-cadherin promoter consistently and dose dependently improved following CoQ_0_ treatment (0.5–2 μM) (Fig. 4b).

The immunofluorescence assay revealed that CoQ0 induced the expression of E-cadherin. E-cadherin antibodies were used for immunofluorescence staining, and the results indicated E-cadherin upregulation in the CoQ_0_-treated cells (Fig. 4c). Western blotting as well as RT-PCR affirmed E-cadherin upregulation by CoQ_0_. Increased dose-dependent expression of Occludin, followed by reduced Vimentin, Slug, Twist, and Snail expression, while an increase expression of E-cadherin was only observed at 0–1 μM and not at 2 μM was recorded for CoQ_0_-treated MDA-MB-231 cells (Fig. 4d). Furthermore, increased mRNA E-cadherin expression and reduced Snail and Slug expression with dose-dependent 6-h CoQ_0_ treatment were observed (Fig. 4e). These outcomes indicate that CoQ_0_ inhibited EMT through the upregulation of E-cadherin pathways.

### CoQ_0_ attenuates EMT by inhibiting Wnt/β-catenin signaling pathways in MDA-MB-231

**Fig. 5:**
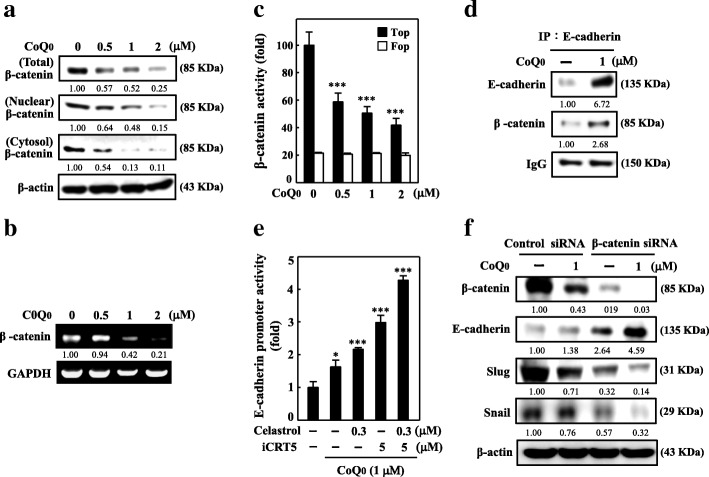
CoQ_0_ inhibits EMT through downregulation of β-catenin signaling pathways in MDA-MB-231 cells. **a** CoQ_0_ inhibited β-catenin nuclear translocation and transcriptional activation. β-catenin levels in total nuclear and cytoplasmic fractions were determined by Western blotting. The cells were incubated with or without CoQ_0_ (0.5–2 μM) for 24 h. Histone H3 and β-actin were used as internal loading controls for nuclear and cytoplasmic fractions, respectively. **b** β-catenin mRNA expression was determined by RT-PCR analyses after 6 h treatment with CoQ_0_ (0.5–2 μM). **c** Transcriptional activity of β-catenin after treatment with CoQ_0_ (0.5–2 μM) for 24 h was monitored by luciferase reporter assay. **d** CoQ_0_ affects E-cadherin through β-catenin. Cells were incubated with or without CoQ_0_ (1 μM) for 24 h. Equivalent amounts of proteins were immunoprecipitated with anti-E-cadherin and anti-β-catenin antibodies and visualized by Western blot analysis. **e** E-cadherin expression was enhanced in cells following treatment with CoQ_0_ (1 μM) in the presence of the NFκB inhibitor celastrol (0.3 μM) and/or β-catenin inhibitor iCRT5 (5 μM) for 24 h. **f** β-catenin gene silencing abolished the CoQ_0_-mediated suppression of EMT. Cells were transfected with a specific β-catenin siRNA or a non-silencing control. Following transfection, the cells were incubated with or without CoQ_0_ (1 μM) for 24 h. Western blot analyses was performed to measure the expression levels of β-catenin transcriptional target genes such as E-cadherin, Slug, and Snail. The results are presented as the mean ± SD of three independent assays. **p < 0.05, ***p < 0.001 significant compared to control cells

E-cadherin/β-catenin protein complexes play an active role in EMT and are critical to cancer progression [34]. To evaluate the effects of CoQ_0_ on the E-cadherin/β-catenin complex; we first measured the β-catenin expression of MDA-MB-231 cells. The results demonstrated that CoQ_0_ treatment suppressed β-catenin expression in the cytoplasm and nucleus dose dependently (Fig. 5a). Moreover, the mRNA gene expression of β-catenin was subdued by CoQ_0_ treatment in the MDA-MB-231 cells (Fig. 5b). To prove if CoQ0 treatment in MDA-MB-231 cells modulated β-catenin transcriptional activity, a TOP/FOP luciferase reporter system was used. Decreased luciferase activity expression was observed in cells transfected with TOP reporter vector when dose dependently treated with CoQ_0_. By contrast, CoQ0 did not affect cells that were transfected with FOP reporter vector and used as negative control. Subsequently, enhanced E-cadherin and β-catenin association was noted (Fig. 5d).

Next, we examined E-cadherin by using a luciferase reporter construct. This construct was stably transfected into MDA-MB-231 cells. A luciferase reporter assay revealed that cells treated with CoQ_0_ (1 μM) caused a profound increase in E-cadherin promoter activity. Intriguingly, cells that underwent pre-treatment with the NFκB inhibitor celastrol (0.3 μM), β-catenin inhibitor iCRT5 (5 μM), or both exhibited a significant increase in E-cadherin luciferase activity relative to the control cells (Fig. 5e), suggesting that CoQ_0_ induces the expression of E-cadherin by suppressing the β-catenin and NFκB signaling pathways in MDA-MB-231 cells. We transfected MDA-MB-231 cells with β-catenin siRNA to confirm this phenomenon; consequently, we observed changes in E-cadherin, Snail, and Slug proteins following CoQ_0_ treatment. Through Western blotting, we determined that CoQ_0_ treatment considerably increased the expression of E-cadherin and suppressed that of Slug and Snail in MDA-MB-231 cells transfected with β-catenin siRNA (Fig. 5e). These results suggest that CoQ0 would act on E-cadherin expression thus modulating EMT by not only Wnt/β-catenin, but also NFκB pathway.

### CoQ_0_ inhibits metastasis and EMT induced by TNF-α/TGF-β

**Fig. 6:**
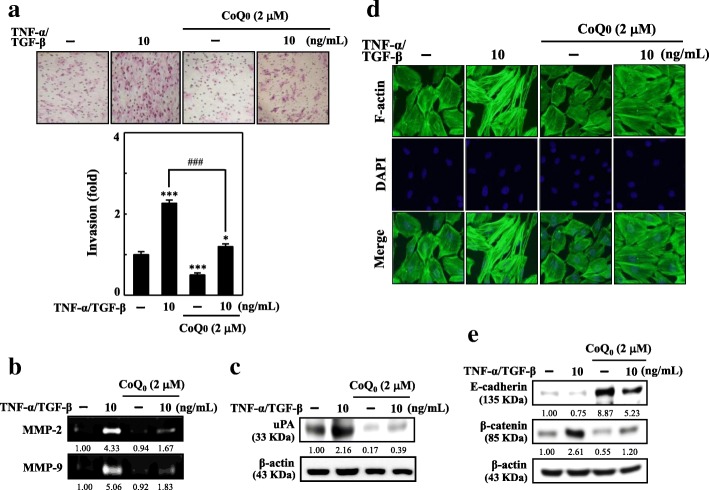
CoQ_0_ inhibits TGF-β/TNF-α-induced metastasis and EMT in MCF-10A cells. Cells were pretreated with 2 μM CoQ_0_ for 1 h and then stimulated with TGF-β/TNF-α (10 ng/mL) for 24 h. (A-C) CoQ_0_ inhibits TNF-α/TGF-β-induced metastasis. **a** Cells invasiveness determined by counting cells in three microscopic fields per sample. **b** CoQ_0_ inhibits TNF-α/TGF-β-induced MMP-2/− 9 and uPA. Inhibition of MMP-2 and MMP-9 activity in conditioned medium from MCF-10A cells was evaluated using gelatin zymography. **c** CoQ_0_ inhibits TNF-α/TGF-β-induced uPA. uPA protein expression was monitored by using Western blot analyses. (D-E) CoQ_0_ inhibits TNF-α/TGF-β-induced EMT. **d** Cytoskeletal pattern of F-actin was measured by immunofluorescence analyses (100 × magnification). **e** CoQ_0_-induced TNF-α/TGF-β decreased E-cadherin and inhibited TNF-α/TGF-β-induced β-catenin. Using Western blot analyses monitored e-cadherin and β-catenin protein expression. The results are presented as the mean ± SD of three independent assays. **p < 0.05, ***p < 0.001 significant compared to control cells; ^##^*p* < 0.01, ^###^*p* < 0.001 significant compared to TNF-α alone treated cells

The non-toxic concentrations of CoQ_0_ (2 μM) were employed to assess the anti-EMT and antimetastatic effects in TNF-α/TGF-β-stimulated non-tumorigenic MCF-10A breast cells. MCF-10A intrusion was ascertained using a Boyden chamber assay. This assay determined cells’ ability to traverse a Matrigel-coated filter’s extracellular matrix layer. The findings revealed that TNF-α/TGF-β treatment enhanced MCF-10A cell invasiveness significantly compared with that of untreated cells. Additionally, CoQ_0_ pre-treatment resulted in considerable inhibition of TNF-α/TGF-β-induced invasiveness of MCF-10A (Fig. 6a). Subsequently, we investigated the effect of CoQ_0_ on TNF-α/TGF-β-induced stimulation of MMPs and uPA levels. Zymography and Western blotting analysis showed that CoQ_0_ pre-treatment attenuated the MMP-2 and MMP-9 activity (Fig. 6b) and uPA protein expression in MCF-10A cells induced by TNF-α/TGF-β (Fig. 6c).

To ascertain the impact of CoQ_0_ on TNF-α/TGF-β-induced cell morphology, MCF-10A cells underwent 24-h TNF-α/TGF-β treatment. Subsequently, their F-actin distribution was analyzed. As shown in Fig. 6d, cells that underwent TNF-α/TGF-β treatment were redistributed from the epithelial to fibroblastic phenotype, whereas CoQ_0_ pre-treatment reversed the TNF-α/TGF-β-induced morphological changes. This result was, as verified through DAPI nuclear staining, independent of apoptosis (Fig. 6d). Notably, our findings confirmed that TNF-α/TGF-β stimulation decreased E-cadherin expression and increased β-catenin expression in MCF-10A cells, a hallmark of EMT (Fig. 6e). Nevertheless, CoQ_0_ pre-treatment increased the TNF-α/TGF-β-induced downregulation of E-cadherin while it downregulated β-catenin (Fig. 6e). These findings confirmed that CoQ_0_ can attenuate EMT by upregulating the E-cadherin and downregulating β-catenin signaling pathways and suppressing metastasis by inhibiting invasion and downregulating the expression of MMP-2/9 and uPA in TNF-α/TGF-β-activated MCF-10A cells.

### CoQ_0_ induces apoptosis through generation of ROS in MDA-MB-231 cells

**Fig. 7:**
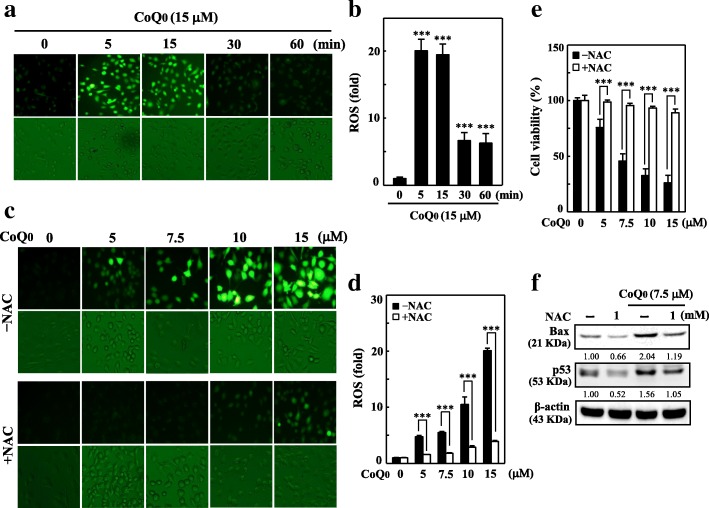
CoQ_0_ induces apoptosis through ROS generation in MDA-MB-231 cells. **a**-**b** CoQ_0_ induced ROS generation. Cells were treated with CoQ_0_ (15 μM) for 0–60 min. The intracellular ROS level as a percentage of the control, is expressed in graphical data. **c**-**e** Cells were pre-treated with antioxidant *N-*acetylcysteine (NAC, 1 mM) for 1 h followed by treatment with CoQ_0_ (5–15 μM for 15 min) and were analyzed for ROS generation (**c** and **d**) and cell viability (**e**). **f** Cells were pre-treated with NAC (1 mM) for 1 h followed by CoQ_0_ (7.5 μM) treatment for 24 h. The expression of Bax and p53 was monitored by Western blot. The results are presented as the mean ± SD of three independent assays. **p < 0.05, ***p < 0.001 significant compared to control cells

Studies have implicated ROS generation as a cellular apoptosis inducer [35]. To ascertain whether the generation of ROS is associated with CoQ_0_-engendered apoptosis, the intracellular ROS level in CoQ_0_-treated MDA-MB-231 cells was determined. CoQ_0_ dose dependently induced apoptosis in breast cancer cells; its effect was observed through death Annexin V-FITC/PI flow cytometry and staining (Additional file 1 a-d). Incubation of cells with CoQ_0_ (15 μM) for 5–60 min caused DCF fluorescence to increase time dependently, which was directly proportionate to the amount of ROS generated maximum at 15 min (Fig. 7a-b). ROS levels dose-dependently increased in CoQ_0_-treated (5–15 μM for 15 min) MDA-MB-231 cells (Fig. 7c-d). However, cells subjected to ROS inhibitor treatment (1 mM NAC for 60 min) before CoQ_0_ treatment exhibited significantly reduced ROS generation (Fig. 7c-d). Another line of evidence revealed that CoQ_0_-induced MDA-MB-231 cell death did not occur in NAC-pre-treated cells (Fig. 7e). Western blot results revealed that NAC preincubation resulted in a gradual decrease in CoQ_0_-induced apoptotic Bax and p53 protein expression (Fig. 7f). These results evince that CoQ_0_ triggered the production of ROS in MDA-MB-231 cells that could engender apoptotic cell death.

### In vivo growth inhibition in xenografted mouse model by CoQ_0_

**Fig. 8:**
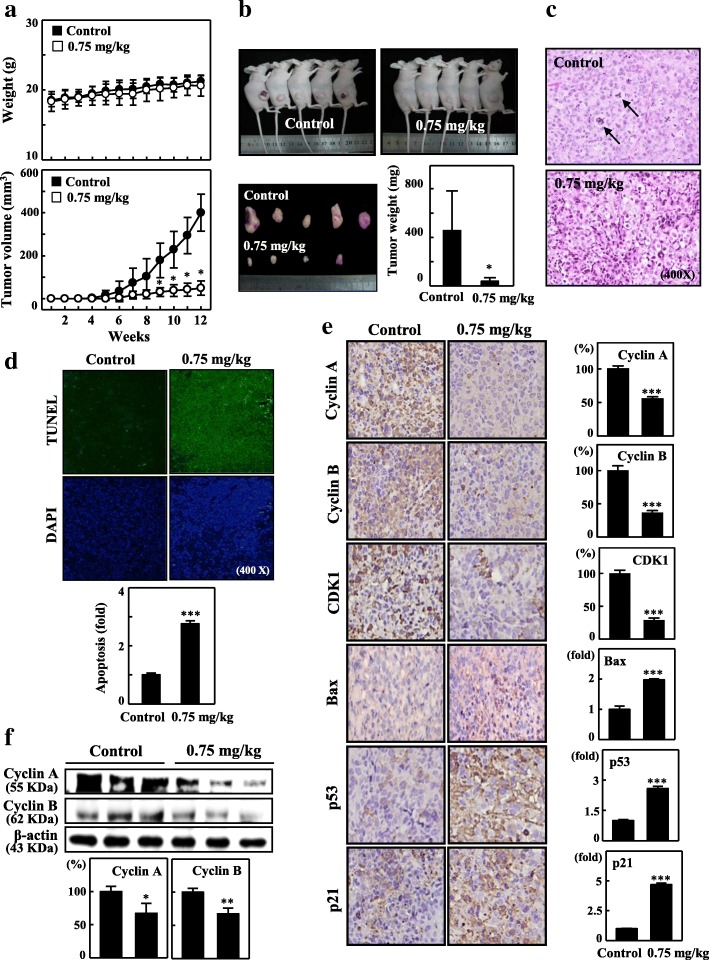
In vivo inhibition of proliferation in mice xenografted with MDA-MB-231 by CoQ_0_. **a** Time-dependent effect of CoQ_0_ on growth of MDA-MB-231 xenografted nude mice was evaluated by measurements of body weight and tumor volume every week. Cells were implanted subcutaneously into the flanks of nude mice on day 0, and animals were subsequently treated with 0.075 mg/kg of CoQ_0_ (three times/week) or vehicle (control). **b** On the 12th week after tumor implantation, the animals were sacrificed and the tumor tissues removed and weighed. The results are presented as the mean ± SE (*n* = 5). **c** Histochemical analysis of proliferation in MDA-MB-231 xenografted tumors. Control and MDA-MB-231 xenografted tumors following CoQ_0_ (0.75 mg/kg) treatments were sectioned, stained with hematoxylin and eosin and examined using light microscopy (400 × magnification). Arrows indicate mitotic cells (tumor control). **d** In-situ apoptosis detection using TUNEL staining in tumor sections from control animals and experimental analogues treated with CoQ_0_ (0.75 mg/kg) (400 × magnifications). The number of apoptotic-positive cells from 3 samples was averaged. **e** Western blotting results demonstrating the effects of CoQ_0_ on the total protein content of Cyclin A and Cyclin B in the xenografted tumors from 3 samples. **f** Xenografted tumor sections were subjected to immunohistochemical analysis for Cyclin A, Cyclin B, CDK1, Bax, p53, and p21. Cells positive for the indicated proteins were counted from 3 fields (200 × magnification) for each tumor sample. Relative changes in protein bands were measured by densitometric analysis, with the control being 100% or 1-fold. The results are the mean (±SE) numbers of cells/microscope field (as percentage) for 5~7 animals per group. Significant at *p < 0.05; **p < 0.01; ***p < 0.001 compared to untreated control cells

To determine the in vivo impact of CoQ_0_ on tumor growth, xenografted nude mice were used. We xenografted MDA-MB-231 cells into them. Observations indicated that all mice were healthy and that their body weights were unaffected during CoQ_0_ treatment (FiA). The MDA-MB-231 xenografted animals were treated with CoQ_0_ (0.75 mg/kg three times/week) or with only vehicle. A significant time-dependent inhibition of tumor volume was observed for CoQ_0_ treatment (Fig. 8a). Additionally, a reduction in tumor weight in the CoQ_0_-treated xenografted mice was observed (Fig. 8b). After 12 weeks, the animals were killed and the xenografted tumor was extracted. Concomitantly, excised tumor sections were observed under the microscope to discern the differences in nuclei and cytoplasmic morphology. The cancer cells in the xenografted mice used as controls appeared large and oval or round in form with myriad nucleoli, and expressed substantial mitotic figure and cellular activity levels (Fig. 8c). By contrast, the CoQ_0_-treated tumor-xenografted mice demonstrated less angiogenesis, and their cells appeared shrunken and condensed. Furthermore, their nuclei exhibited karyopyknosis, implying carcinoma regression or activity (Fig. 8c). These results suggest that CoQ0 promotes antitumor activity in xenografted mouse models.

### Fragmentation of apoptotic DNA induced by CoQ_0_ in tumor-xenografted mouse model

CoQ_0_’s role in apoptosis in MDA-MB-231-xenografted mice was ascertained using the TUNEL assay on cancerous sections. The presence of a greater number of TUNEL-positive cells in mouse tumors treated with CoQ_0_ compared with those of the untreated controls (*p* < 0.001) suggested that CoQ_0_ treatment was affiliated with reduced cell proliferation and increased apoptosis (Fig. 8d). In addition, immunohistochemical analysis revealed that CoQ_0_-treated mice significantly decreased Cyclin A, Cyclin B, and CDK1 expression and increased Bax, p53, and p21 expression in MDA-MB-231 xenografted tumor tissues (Fig. 8e). By contrast, CoQ_0_ treatment in MDA-MB-231 cells resulted in G2/M arrest and downregulation of the expression of Cyclin B, Cyclin A, Cdc2, and Cdc25C (Additional file 2 a-b). Subsequently, Western blotting was employed to determine the effect of CoQ_0_ on Cyclin A and B proteins in MDA-MB-231 xenografted tumor tissues. Cyclin A and B expression was suppressed relative to that the controls (Fig. 8f). In summary, the inhibition of tumor development by induction of apoptosis in TNBCs during CoQ_0_ treatment was observed.

### CoQ_0_ attenuates in vivo lung metastasis

**Fig. 9:**
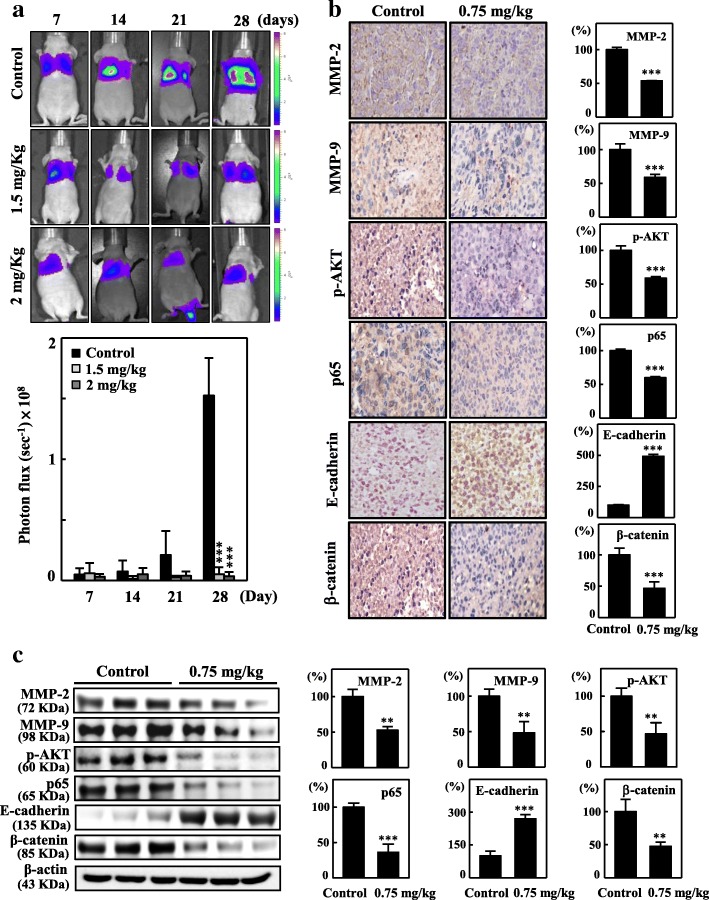
In vivo anti-metastatic activity of CoQ_0_. **a** CoQ_0_ inhibited lung metastasis in living MDA-MB-231-luciferase-injected mice by bioluminescence imaging. Mice were treated with CoQ_0_ (1.5 or 2 mg/kg) and then the MDA-MB-231-luciferase cells (1 × 10^6^ cells/well) were intravenously injected. The mice were anaesthetized, and luciferin was intraperitoneally injected. The mice were imaged using the IVIS 200 system, and the photons from the whole animal were quantified. Diagrams showing the bioluminescent signal emitted from the whole body. The color overlay on the image represents the luminescence (photons/sec) emitted from the animal, as indicated by the color scales. Photos are representative images (*n* = 4). **b**-**c** Tumor sections were from control animals and experimental analogues treated with CoQ_0_ (0.75 mg/kg). **b** MMP-2, MMP-9, p-AKT, p65, E-cadherin, and β-catenin were examined using immunohistochemical staining. **c** MMP-2, MMP-9, p-AKT, p65, E-cadherin, and β-catenin were examined using Western blotting. The results are the mean (±SE) numbers of cells/microscope field (as percentage) for 3 animals per group. Western blotting results showing the effects of CoQ_0_ on the cumulative protein content in the xenograft tumors. β-actin were used as an internal control. Relative changes in protein bands were measured by densitometric analysis with the control being 100%. The results are the mean (±SE) numbers of cells/microscope field (as percentage) for 5~7 animals per group. Significant at *p < 0.05; **p < 0.01; ***p < 0.001 compared to untreated control cells

To validate the potential effect of CoQ_0_ on metastasis, we treated mice with CoQ_0_ (1.5 or 2 mg/kg) and then intravenously injected them with MDA-MB-231-luciferase cells (1 × 10^6^ cells/well). The luciferase-labeled MDA-MB-231 cancer cells metastasized to the lungs after 28 d in the control mice (Fig. 9a). CoQ_0_ treatment blocked MDA-MB-231 cell lung metastasis to a statistically significant level; photon flux in the mouse lungs fell by > 90% in the CoQ_0_ treatment groups (1.5 and 2 mg/kg) (Fig. 9a). These findings imply that CoQ_0_ entirely suppresses highly metastatic breast cancer cell lung metastasis at concentrations of 1.5 or 2 mg/kg.

To validate the mechanism by which CoQ_0_ attenuates tumor metastasis, the metastasis-related proteins from control and CoQ_0_-treated (0.75 mg/kg) mouse tumors were studied. Immunohistochemical and Western blot results revealed that CoQ_0_ inhibited MMP-9, MMP-2, p-AKT, p65, and β-catenin expression and increased E-cadherin expression relative to the controls (Fig. 9b-c). Comprehensive results confirmed that CoQ_0_ suppresses metastasis by downregulation of MMP-2, MMP-9, p65, β-catenin, and p-AKT and upregulation of E-cadherin proteins. Furthermore, the gene expression patterns of MMP-2 and MMP-9 mRNA were substantially suppressed by CoQ_0_ (Additional file 3 a). In addition, immunohistochemical analysis revealed significant decreases in uPA, uPAR, Vimentin, COX-2, Twist, VEGF, and p-mTOR in CoQ_0_-treated mice (Additional file 3 b). Subsequently, we investigated the effects of CoQ_0_ on EMT regulatory proteins. CoQ_0_ substantially reduced uPA, uPAR, COX-2, and Vimentin expression and promoted Occludin protein levels compared with those of the control group as discerned through Western blot analysis (Additional file 3 c). These results indicate that CoQ_0_ attenuated metastasis and EMT in MDA-MB-231-xenografted nude mice.

## Discussion

EMT is a physiological process that is usually activated during wound healing and embryonic development. It is a crucial step in cancerous metastatic progression [36]. During EMT, the epithelial-derived tumor cells stimulate intercellular and intracellular changes that contribute to mesenchymal cell phenotypes, including cytoskeleton reorganization, polarity alteration, extracellular matrix remodeling, and migratory ability acquisition [12]. Numerous researchers have investigated EMT’s role in breast cancer. Mesenchymal EMT molecular marker overexpression in biopsies of breast cancer is correlated with increased recurrence, adverse clinicopathological characteristics, reduced survival, and tumor aggressiveness [37]. Therefore, efficacious therapeutic strategies must be established to reduce breast cancer cell tumor aggressiveness and prevent malignant growth. In our previous study, we reported that CoQ_0_ exerts antimetastatic effects in melanoma carcinomas. This action may be because of the modulation of the Wnt/β-catenin signaling pathway in B16F10 melanoma cells [24]. In the current study, CoQ_0_’s antimetastatic and anti-EMT abilities were characterized, and mechanisms responsible for its effects in MDA-MB-231 were studied. Additionally, E-cadherin downregulation and alterations of the EMT-linked signaling regulator indicate that MDA-MB-231 cells can commence and propagate the EMT process in cancer cells. The salubrious impact of pre-treatment with CoQ_0_ was proven by the renewal of E-cadherin protein and transcriptional activity. The renewal of E-cadherin was linked to β-catenin, NFκB, and MMP-9 inhibition, a key molecular event in EMT inhibition. Increased cancer cell migration and invasion, mammosphere formation, colony formation ability, and tumor growth were effectively suppressed through CoQ_0_ treatment. These findings indicate that CoQ_0_ is an antimetastatic and anti-EMT substance, and the potential molecular signaling pathways that are involved in this process can be inferred.

E-cadherin is an adherens junction protein expressed in normal breast tissue; it is a useful phenotypic marker in cases of breast cancer [38]. In this study, the transcriptional activity and protein levels of E-cadherin were investigated to ascertain the manifestation of EMT with TNF-α/TGF-β-stimulation in TNBC cells. The results of immunofluorescence and luciferase activity and Western blotting revealed that TNF-α/TGF-β could undermine E-cadherin junctions by governing the organization of actin in MDA-MB-231 cells. These results were supported by evidence from previous studies [33]. E-cadherin loss stimulates EMT, which plays a major role in the development of carcinomas to a metastatic state. Although the mechanism that is involved in E-cadherin inactivation in cancer cells remains vague, alterations of transcriptional levels may explain its downregulation [39]. Therefore, an effective strategy for controlling metastasis and EMT progression may be restoring or preventing E-cadherin downregulation by using TNF-α/TGF-β. In this work, the restoration of E-cadherin protein levels and transcriptional activity through CoQ_0_ treatment inhibited EMT and the associated carcinoma metastasis. Restoration of E-cadherin expression at the transcription and protein level by Withaferin A was linked to metastasis and cell proliferation inhibition in breast cancer cells [33]. Furthermore, our in vivo study proved that CoQ_0_ significantly increased the expression of E-cadherin in MDA-MB-231 tumors and prevented MDA-MB-231 cell lung metastasis, implying that CoQ_0_ arrests EMT programming because of its antimetastatic properties in breast cancer cells.

EMT is a crucial mechanism in cancer development and in the first phase of metastasis. Retardation of E-cadherin/β-catenin may facilitate tumor invasion and metastasis [40]. Increasingly, evidence indicates that E-cadherin has a vital function in β-catenin function and stabilization. When E-cadherin expression decreased, β-catenin was able to be separated from the E-cadherin/β-catenin complexes and could translocate to the nucleus freely. Moreover, β-catenin bound with the TCF/LEF-1 element after which it activated certain promigratory genes required for EMT combined with related transcription factors [41]. Some transcription factors, including Slug and Snail, which are both among the transcriptional targets of β-catenin, may be associated with E-cadherin and EMT repression [42]. In this work, CoQ_0_ induced E-cadherin and significantly decreased nuclear β-catenin, Snail, and Slug protein association, as shown by Western blot analysis. Furthermore, immunoprecipitation assays revealed that CoQ_0_ increased E-cadherin and β-catenin expression relative to that of the untreated group. This result indicates that CoQ_0_ may restore the formation of E-cadherin/β-catenin complexes in MDA-MB-231 cells, impeding nuclear transport of β-catenin to a greater extent, which subsequently enhances the expression of E-cadherin by inhibiting Slug. Our results are a strong indication notion that the anti-EMT impact of CoQ_0_ is correlated with the governance of the formation of E-cadherin/β-catenin complexes.

Matrix metalloproteases (MMPs) have a key function in extracellular matrix (ECM) remodeling and degradation [43]. MMPs play roles in all stages of breast carcinogenesis, from tumor initiation to metastasis. Among the several MMP family members, MMP-2 and MMP-9 were highly expressed in invasive breast cancer cells [44]. The present study determined that CoQ_0_ pre-treatment abrogated the TNF-α/TGF-β-induced MMP-9 and MMP-2 expression levels in MDA-MB-231 cells. Therefore, MMP-9 and MMP-2 could be CoQ_0_-responsive mediators whose ECM degradation could result in ensuing cancer invasion and migration.

The MMP-9 promoter region possesses cis-regulatory elements, such as two AP-1 and one NFκB binding sites. These sites are not present in MMP-2’s promoter region [45]. Therefore; we investigated the effects of CoQ_0_ on NFκB, which plays a major role in the transcription of MMP-9. NFκB activation results in cell invasion, metastasis, and survival advantages and drug resistance to several cancer types [46]. Nuclear translocation and transcriptional activation of NFκB subunits are strictly governed by NFκB’s inhibitory protein, I-κBα, whose phosphorylation releases NFκB subunits [47]. The data from our experiments clearly demonstrate that treatment with CoQ_0_ suppressed the transcriptional activation and nuclear translocation of NFκB. This effect may have been caused by the inhibition of I-κB kinase phosphorylation and I-κBα degradation. Furthermore, pre-treatment with celastrol remarkably reduced the expression of MMP-9 and NFκB proteins. This result indicates that the NFκB pathway is the principal regulatory pathway in the suppression of MMP-9 expression by treatment with CoQ_0_.

PI3K/AKT is the major pathway for tumor invasion [48]. Therefore, we sought to determine whether CoQ_0_ suppresses the phosphorylation of PI3K/AKT because its signaling cascade is the main component upstream of NFκB and plays a key role in cellular adhesion, differentiation, and growth. The PI3K/AKT axis plays a principal role in metastasis and tumor invasion through activation of NFκB-mediated MMP-9 [49]. Our findings clearly demonstrate that CoQ_0_ treatment suppressed PI3K/AKT phosphorylation substantially. Furthermore, NFκB activation and MMP-9 were significantly reduced by blockage of the PI3K/AKT pathway with LY249002 treatment. These findings reveal that CoQ_0_ lowers the expression of MMP-9 by blocking NFκB activation through PI3K/AKT and thus suppresses MMP-9-mediated cell intrusion in MDA-MB-231 human breast cancer cells. Our findings are consistent with those of a report that suggested that LFG-500 extracted from flavonoid inhibits cancer cell intrusion by suppressing the PI3K/AKT/NFκB/MMP-9 signaling pathways [49].

The present paper documents the anti-EMT and antimetastatic capabilities of CoQ_0_ and lists the mechanisms that may cause its effects in non-tumorigenic MCF-10A cells under stimulation induced by TNF-α/TGF-β. TGF-β enhances tumor development by activating EMT. TGF-β-induced EMT exhibited the following attributes: the loss of junctional E-cadherin localization, acquisition of fibroblastic morphology, and increased cellular motility [50]. TNF-α is a proinflammatory cytokine and plays a vital role in tumor malignancy, including motility, tumor cell invasion, and metastasis [51]. TNF-α induced EMT in renal cell carcinoma by suppressing E-cadherin expression and promoting Vimentin and MMP-9 protein expression [52]. Stimulation of TGF-β, TNF-α, or both may cause an EMT-like phenomenon, E-cadherin expression reduction, and morphological changes in Madin–Darby canine kidney cells [53]. In the present study, downregulation of E-cadherin, upregulation of β-catenin, and changes in EMT-linked signaling regulators initiated and propagated EMT in MCF-10A cells stimulated using TNF-α/TGF-β. The advantageous impact of pre-treatment with CoQ_0_ was based on the renewal of transcriptional and E-cadherin promoter activity against losses induced by TNF-α/TGF-β. Additionally, the renewed E-cadherin promoter activity was linked to β-catenin, NFκB, and MMP-2/− 9 inhibition, which is a vital molecular event in the inhibition of EMT induced by TNF-α/TGF-β.

Human mammary epithelial cells forms numerous non-adherent spherical colonies know as, “non-adherent mammospheres”. [54]. They comprise of several stem cells that regenerate to form mammospheres within serial passages and progenitor cells that perform multi origin differentiation. Accumulating evidence suggests that many cancers, including breast cancer, are guided by a cellular subpopulation, designated as cancer stem cells (CSCs), that mediates tumor metastasis and resistance to conventional therapies. Thus, preventing CSC growth in breast cancer is the optimal strategy for inhibiting tumor development and metastasis [55]. Therefore, research on CoQ_0_-induced molecular mechanisms that mediate CSC proliferation is vital to clarify CoQ_0_’s antimetastatic and anticancer activities. Our study revealed that CoQ_0_ treatment considerably lowered mammosphere formation and sphere size. These results suggest that CoQ_0_ inhibits mammosphere formation.

Apoptosis induction, restriction of cell proliferation by chemical or biological agents, and cell-cycle arrest are intended to be effective strategies in cancer management, particularly of TNBCs. Apoptosis-inducing agents are under investigation as alternative tools for cancer treatment management. A study reported that CoQ_0_ treatment caused the proportion of late apoptotic MDA-MB-231 cells to rise when Annexin V/PI staining and then flow cytometry were employed [56]. In the present study, the treatment of MDA-MB-231 cells with CoQ_0_, successfully inhibited anchorage-independent growth and cell proliferation. Examples of the characteristic features of apoptosis are chromatin condensation, internucleosomal DNA cleavage, caspase activation, and cellular morphological changes [57]. In the current study, we demonstrated that, by treating MDA-MB-231 cells with CoQ_0_, apoptotic cell death linked to DNA fragmentation increased considerably. In a study, treating human lung cancer cells with CoQ_0_ increased the number of early and late apoptotic cells and reduced apoptotic cell death through antioxidant treatment [19]. Other studies have demonstrated that methoxy-containing analogs of CoQ_0_ and quinones that have similar structures to CoQ_0_ have a cytotoxic influence on human cancer cells because they induce apoptosis [58]. Researchers employed various CoQ analogs and recorded enhanced DNA fragmentation, caspase-3 activation, and apoptosis for CoQ_4_ and CoQ_2_ in HL 60 human leukemia cells. However, these effects were not observed for CoQ_10_ or CoQ_6_. These results suggest that CoQ_0_ analogs pro-apoptotic and anticancer attributes vary depending on the location of the methoxy-substitutions on the quinone nucleus and the length of the isoprenyl side chain. No matter the cell line, CoQ_0_, which possesses no isoprenoid units, suppresses cancer cell growth and triggers early and late apoptosis.

The excessive generation of ROS can induce cell-cycle arrest, oxidative stress, damaged DNA in cancer cells, cell function loss, and cellular apoptosis [59]. CoQ_0_’s favorable impact on breast cancer cell lines is linked to mitochondrial dysfunction and the overproduction of intracellular ROS. ROS causes the mitochondrial permeability transition pore to open, mitochondrial proapoptotic factors to be released, and the mitochondrial membrane to depolarize during mitochondria-mediated apoptosis [60]. The present study determined that CoQ_0_ treatment leads to a notable increased in intracellular ROS production in MDA-MB-231 cells. By contrast, the antioxidant, NAC, inhibited ROS production, which reduced apoptosis significantly, indicated that MDA-MB-231-cell apoptosis induced by CoQ_0_ had a close link with ROS production. CoQ_0_ potentially play roles as upstream signaling molecules to induce cell apoptosis mediated by mitochondria. Our findings are agreement with those of prior investigations indicating that natural compounds (e.g., celastrol and deltonin) induce MDA-MB-231 cell ROS-mediated mitochondrial apoptosis [61].

Disruption of the cancer cell cycle is a therapeutic objective of research on novel cancer drugs. This is linked to lower Cyclin A, Cyclin B, Cdc2, and Cdc25C expression and higher CDK inhibitor p21 expression. In eukaryotes, cell-cycle progression included the resultant triggering of CDKs; their activation is cyclin associated. Among CDKs, Cdc2 and CDK2 kinases are mainly triggered with Cyclin B and Cyclin A during G2/M phase progression [62]. Cdc2/Cyclin A and Bi kinase complex activity was suppressed by phosphorylating Tyr15 of Cdc2. Cdc25C phosphatase catalyzed the dephosphorylation of Tyr15 of Cdc2. This reaction was considered the rate-limiting step in their progression into mitosis [63]. P21 might facilitate G2/M cell-cycle arrest maintenance through CyclinB1/Cdc2 complex inactivation, thereby disrupting the cell nuclear antigen–Cdc25C interaction [64]. The findings suggest that Cdc25C, Cdc2, Cyclin A, and Cyclin B expression is downregulated, and the CDK inhibitor p21 increased in MDA-MB-231 cells treated with CoQ_0_, which arrests G2/M phase. The present study’s data suggest that the monitored suppression of MDA-MB-231 cell proliferation linked to CoQ_0_ treatment was because of G2/M-phase cell-cycle arrest and not G1 arrest. Intriguingly, our results differ from those of the previous report that indicated that treatment of MDA-MB-231 cells with CoQ_0_ led to G0/G1-phase cell-cycle arrest.

To enhance CoQ_0_’s antimetastatic and anticancer attributes, an in vivo investigation of CoQ_0_-treated MDA-MB-231-xenografted nude mice was executed. CoQ_0_-treated xenografted nude mice resulted in a significant fall in tumor volume and significantly prevented lung metastasis. The observed anticancer action is seemingly related to mitotic cell inhibition and substantial proliferation of apoptotic cells in tumors treated with CoQ_0_. Fewer mitotic-positive cells in the HL60 xenografted nude mouse tumors treated with CoQ0 indicated reduction in cell proliferation. This reduced cell proliferation may cause a lower tumor volume in nude mice [65]. Furthermore, antimetastatic activity may be linked to the upregulation of E-cadherin and the downregulation of MMP-2, MMP-9, p-AKT, p65, and β-catenin proteins. These in vivo results verify CoQ_0_’s effective antimetastatic and antitumor attributes against TNBC that are in agreement with its in vitro anticancer attributes.

## Conclusion

This work determined for the first time that non-cytotoxic concentrations of CoQ_0_ exhibit antimetastatic and anti-EMT attributes in MDA-MB-231 breast cancer cell. EMT inhibition in MDA-MB-231 cells was linked to the renewal of transcriptional activity and E-cadherin protein. CoQ_0_ inhibited mammosphere formation and exhibited its anti-invasive activity by downregulating the PI3K/AKT/NFκB/MMP-9 signaling pathways. Furthermore, CoQ_0_ induced ROS-mediated apoptosis and significantly inhibited the growth of tumors in MDA-MB-231 xenografted nude mice. Our results provide new insight into the potential molecular mechanisms that underlie CoQ_0_’s promising anticancer attributes. Our findings justify further preclinical and clinical assessments of CoQ_0_ for metastatic breast cancer therapy.

## Additional files

Additional file 1: CoQ_0_-induced apoptosis in MDA-MB-231 cells. The cells were exposed to CoQ_0_ (5–15 μM for 24 h). (**a**-**b**) The TUNEL assay was performed to determine apoptotic DNA fragmentation. The green florescence indicates the number of TUNEL positive cells in the microscopic fields (400 × magnification) from three separate samples. The percentage of apoptotic cells was calculated by measuring the florescence intensity of treated cells using commercially available software. (**c**) Annexin V-FITC and PI staining was used to identify the early/late apoptosis or necrosis, and the data were analyzed using flow cytometry. The results in each quadrant are labeled and interpreted as follows: (Q1) PI positive, Annexin V-FITC-negative stained cells/necrosis. (Q2) PI positive, Annexin V-FITC-positive stained cells/late apoptosis. (Q3) Cells negative for both PI and Annexin V-FITC staining/normal live cells. (Q4) PI-negative, Annexin V-FITC-positive stained cells/early apoptosis. (**d**) Effects of CoQ_0_ on apoptotic-related proteins. Protein levels of mitochondria/cytosolic cytochrome c, caspases-9, caspase-3, and PARP, Bax, Bcl-2, and p53 were analyzed by Western blotting. The results are presented as the mean ± SD of three independent assays. ***p < 0.001 significant compared to control cells. (PPTX 51519 kb)

Additional file 2: CoQ_0_ treatment induces G_2_/ M cell-cycle arrest in MDA-MB-231 cells. (**a**) Cells were treated with CoQ_0_ (5–15 μM) for 24 h, stained with PI and analyzed for cell-cycle phase using flow cytometry. The cellular distributions (%) in different phases of the cell cycle (sub-G_1_, G_1_,S and G_2_/M) were determined after treatment with AS. The flow cytometry graph shown here is from one representative experiment that was performed in triplicates. (**b**) The effects of CoQ_0_ AS on cell-cycle regulatory proteins. HL-60 cells were treated with increasing concentrations of CoQ_0_ (5–15 μM) for 24 h. Cell-cycle regulatory proteins, including Cyclin A, Cyclin B, p21, Cdc2, Cdc25C, CDK2, and CDK4 were examined using Western blot analyses. The results are presented as the mean ± SD of three independent assays. **p < 0.05, ***p < 0.001 significant compared to control cells. (PPTX 4253 kb)

Additional file 3: Metastasis and EMT inhibition by CoQ_0_ in MDA-MB-231 xenografted tumors. Tumor sections were from control animals and experimental analogues treated with CoQ_0_ (0.75 mg/kg). (**a**) Cells positive for the indicated proteins were counted from 3 fields (200 × magnification) for each tumor sample, and MMP-2 and MMP-9 were examined using RT-PCR. (**b**) uPA, uPAR, Vimentin, COX-2, Twist, VEGF, and p-mTOR were examined using immunohistochemical staining. (**c**) uPA, uPAR, Vimentin, COX-2, and Occludin were examined using Western blotting. Western blot on the effects of CoQ_0_ on the total protein contents in the xenograft tumors. β-actin was used as the control. Relative changes in protein bands were measured by densitometric analysis with the control being 100%. The results are the mean (±SE) numbers of cells/microscope field (as percentage) for 3 animals per group. Significant at *p < 0.05; **p < 0.01; ***p < 0.001 compared to untreated control cells. (PPTX 12155 kb)

## Abbreviations

EMT: Epithelial–mesenchymal transition
HER2: Human epidermal growth factor receptor 2
TNBC: Triple Negative Breast Cancer Cell
