# Correction: Anti-EMT properties of CoQ0 attributed to PI3K/AKT/NFKB/MMP-9 signaling pathway through ROS-mediated apoptosis

**DOI:** 10.1186/s13046-026-03654-1

**Published:** 2026-02-14

**Authors:** Hsin-Ling Yang, Varadharajan Thiyagarajan, Pei-Chun Shen, Dony Chacko Mathew, Kai-Yuan Lin, Jiunn-Wang Liao, You-Cheng Hseu

**Affiliations:** 1 Institute of Nutrition, College of Biopharmaceuticals and Food Sciences, China Medical University, Taichung, 40402 Taiwan; 2 Department of Cosmeceutics, College of Biopharmaceutical and Food Sciences, China Medical University, No. 91, Hsueh-Shih Road, Taichung, 40402 Taiwan; 3 Department of Medical Research, Chi-Mei Medical Center, Tainan, 710 Taiwan; 4 Institute of Veterinary Pathology, National Chung Hsing University, Taichung, 40227 Taiwan; 5 Department of Health and Nutrition Biotechnology, Asia University, Taichung, 41354 Taiwan; 6 Chinese Medicine Research Center, China Medical University, Taichung, 40402 Taiwan; 7 Research Center of Chinese Herbal Medicine, China Medical University, Taichung, 40402 Taiwan

**Correction: J Exp Clin Cancer Res 38**,** 186 (2019)**

**https://doi.org/10.1186/s13046-019–1196-x**

Following publication of the original article [1], the authors found that Figures 4a, 6d, and 9b displayed the morphological changes assessed using microscopy. The image panels were carelessly created by one of the authors using representative images from a collection of observed images, which led to overlap or duplication in a few instances.

**Incorrect** Fig. 4a

**Fig. 4:**
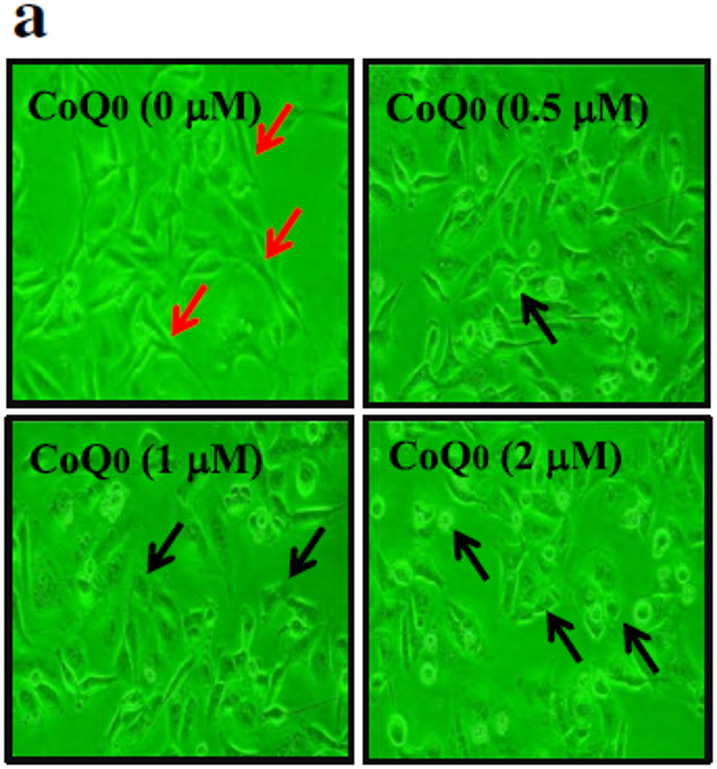
CoQ_0_ inhibits EMT through up-regulation of E-cadherin signaling pathways in MDA-MB-231 cells. **a-d** Cells were treated with CoQ_0_ (0.5–2 µM) for 24 h. **a** Morphological changes were examined by phase-contrast microscope (200× magnification). **b** Transcriptional activity of E-cadherin was monitored by luciferase reporter assay. c Immunofluorescence analysis for E-cadherin protein expression. d CoQ0-induced modulation of epithelial (E-cadherin and Occludin) and mesenchymal marker proteins (Vimentin, Slug, Twist, and Snail) were monitored using Western blot analyses. e mRNA expression of E-cadherin, Vimentin, Slug, and Snail after 6 h treatment with CoQ0 (0.5–2 µM) was measured by RT-PCR analyses. As internal control GAPDH was used. The results are presented as the mean ± SD of three independent assays. **p < 0.05, ***p < 0.001 significant compared to control cells

**Correct** Fig. 4a

**Fig. 4:**
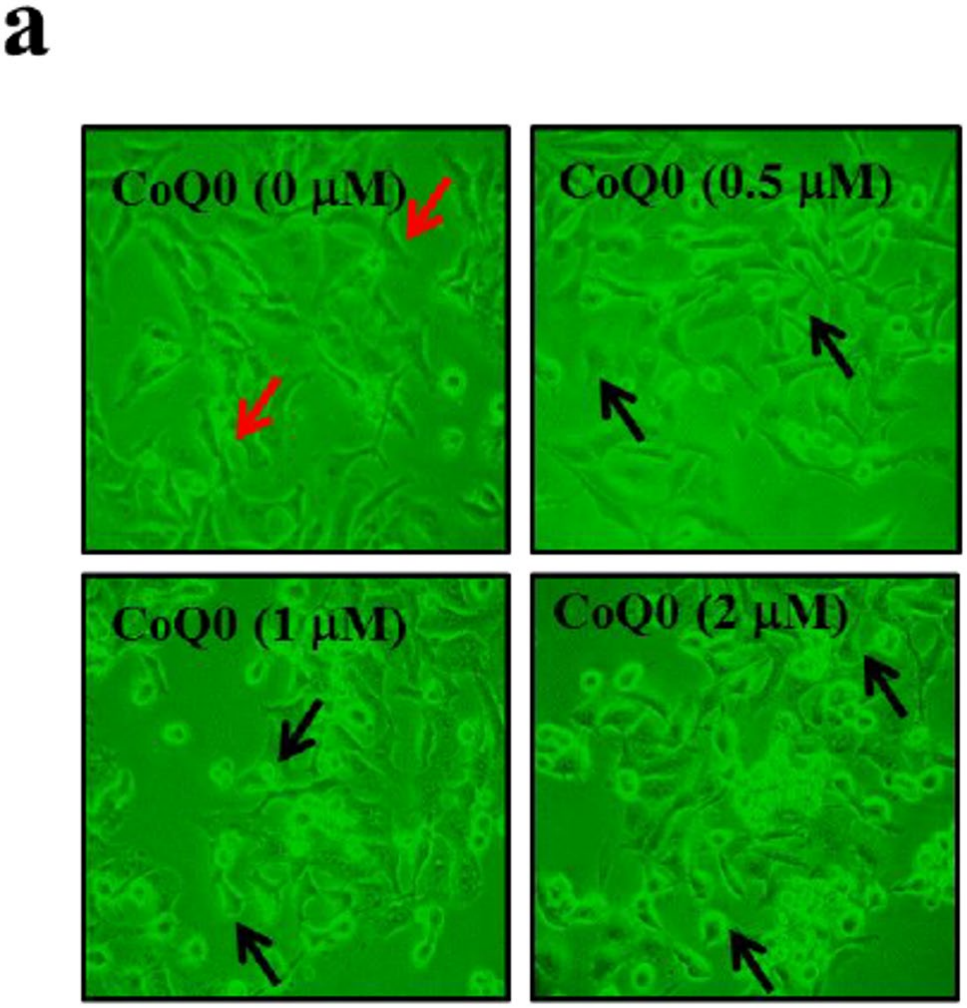
CoQ_0_ inhibits EMT through up-regulation of E-cadherin signaling pathways in MDA-MB-231 cells. **a-d** Cells were treated with CoQ_0_ (0.5–2 µM) for 24 h. **a** Morphological changes were examined by phase-contrast microscope (200× magnification). **b** Transcriptional activity of E-cadherin was monitored by luciferase reporter assay. c Immunofluorescence analysis for E-cadherin protein expression. d CoQ0-induced modulation of epithelial (E-cadherin and Occludin) and mesenchymal marker proteins (Vimentin, Slug, Twist, and Snail) were monitored using Western blot analyses. e mRNA expression of E-cadherin, Vimentin, Slug, and Snail after 6 h treatment with CoQ0 (0.5–2 µM) was measured by RT-PCR analyses. As internal control GAPDH was used. The results are presented as the mean ± SD of three independent assays. **p < 0.05, ***p < 0.001 significant compared to control cells

**Incorrect** Fig. 6d

**Fig. 6:**
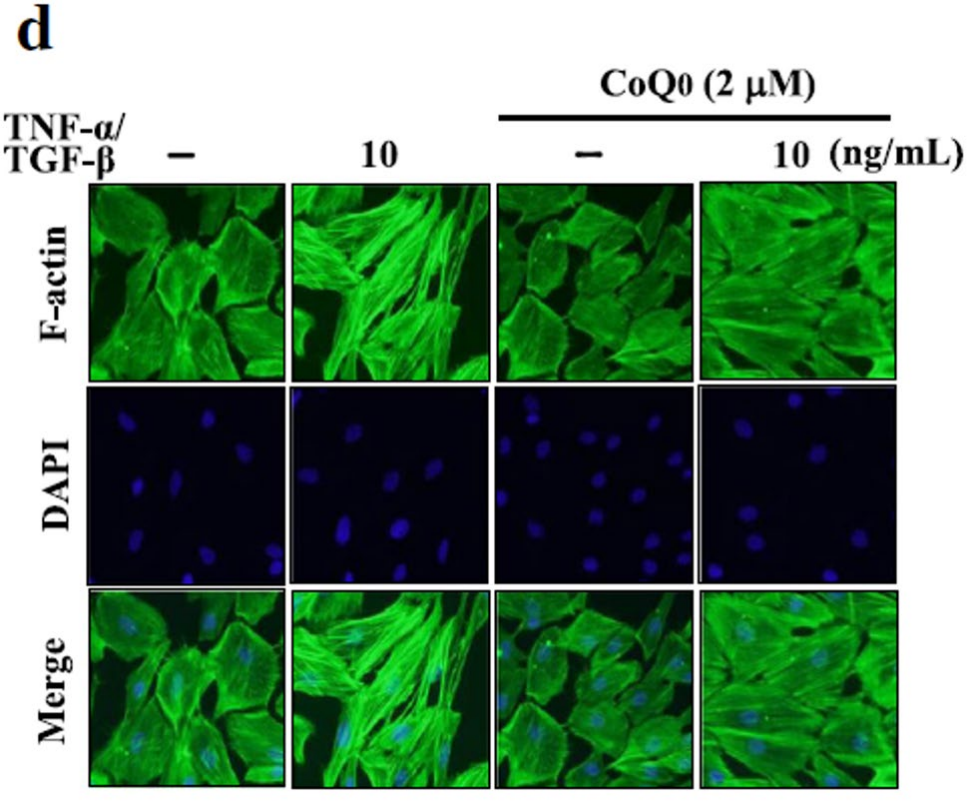
CoQ_0_ inhibits TGF-β/TNF-α-induced metastasis and EMT in MCF-10 A cells. Cells were pretreated with 2 µM CoQ_0_ for 1 h and then stimulated with TGF-β/TNF-α (10 ng/mL) for 24 h. (A-C) CoQ_0_ inhibits TNF-α/TGF-β-induced metastasis. **a** Cells invasiveness determined by counting cells in three microscopic fields per sample. b CoQ0 inhibits TNF-α/TGF-β-induced MMP-2/− 9 and uPA. Inhibition of MMP-2 and MMP-9 activity in conditioned medium from MCF-10 A cells was evaluated using gelatin zymography. c CoQ0 inhibits TNF-α/TGF-β-induced uPA. uPA protein expression was monitored by using Western blot analyses. (D-E) CoQ0 inhibits TNF-α/TGF-β-induced EMT. d Cytoskeletal pattern of F-actin was measured by immunofluorescence analyses (100 × magnification). e CoQ0-induced TNF-α/TGF-β decreased E-cadherin and inhibited TNF-α/TGF-β-induced β-catenin. Using Western blot analyses monitored e-cadherin and β-catenin protein expression. The results are presented as the mean ± SD of three independent assays. **p < 0.05, ***p < 0.001 significant compared to control cells; ##p < 0.01, ###p < 0.001 significant compared to TNF-α alone treated cells

**Correct** Fig. 6d

**Fig. 6:**
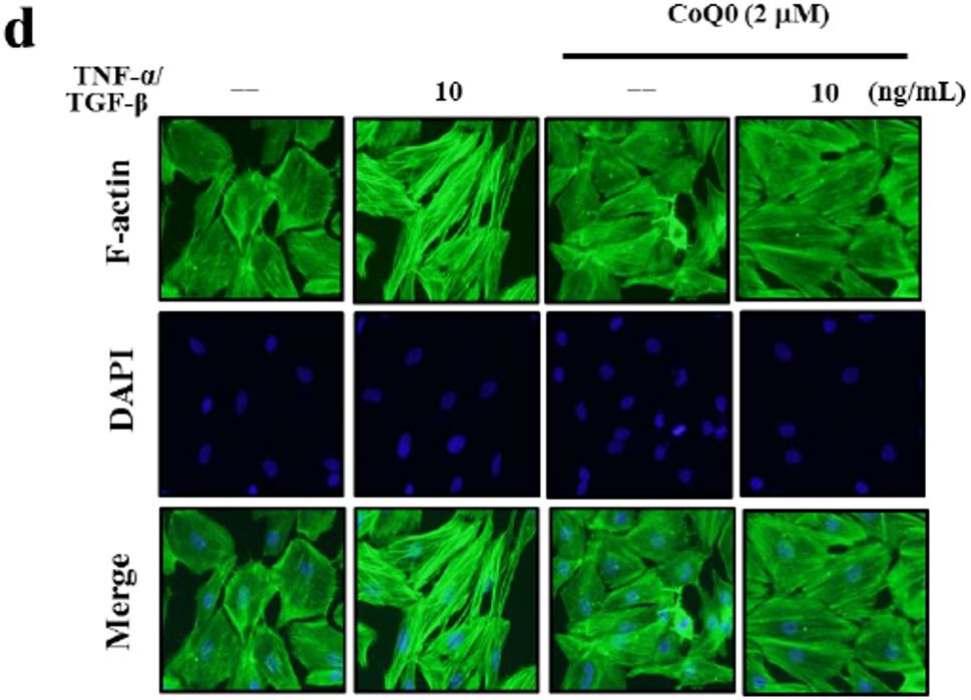
CoQ_0_ inhibits TGF-β/TNF-α-induced metastasis and EMT in MCF-10 A cells. Cells were pretreated with 2 µM CoQ_0_ for 1 h and then stimulated with TGF-β/TNF-α (10 ng/mL) for 24 h. (A-C) CoQ_0_ inhibits TNF-α/TGF-β-induced metastasis. **a** Cells invasiveness determined by counting cells in three microscopic fields per sample. b CoQ0 inhibits TNF-α/TGF-β-induced MMP-2/− 9 and uPA. Inhibition of MMP-2 and MMP-9 activity in conditioned medium from MCF-10 A cells was evaluated using gelatin zymography. c CoQ0 inhibits TNF-α/TGF-β-induced uPA. uPA protein expression was monitored by using Western blot analyses. (D-E) CoQ0 inhibits TNF-α/TGF-β-induced EMT. d Cytoskeletal pattern of F-actin was measured by immunofluorescence analyses (100 × magnification). e CoQ0-induced TNF-α/TGF-β decreased E-cadherin and inhibited TNF-α/TGF-β-induced β-catenin. Using Western blot analyses monitored e-cadherin and β-catenin protein expression. The results are presented as the mean ± SD of three independent assays. **p < 0.05, ***p < 0.001 significant compared to control cells; ##p < 0.01, ###p < 0.001 significant compared to TNF-α alone treated cells

**Incorrect** Fig. 9b

**Fig. 9:**
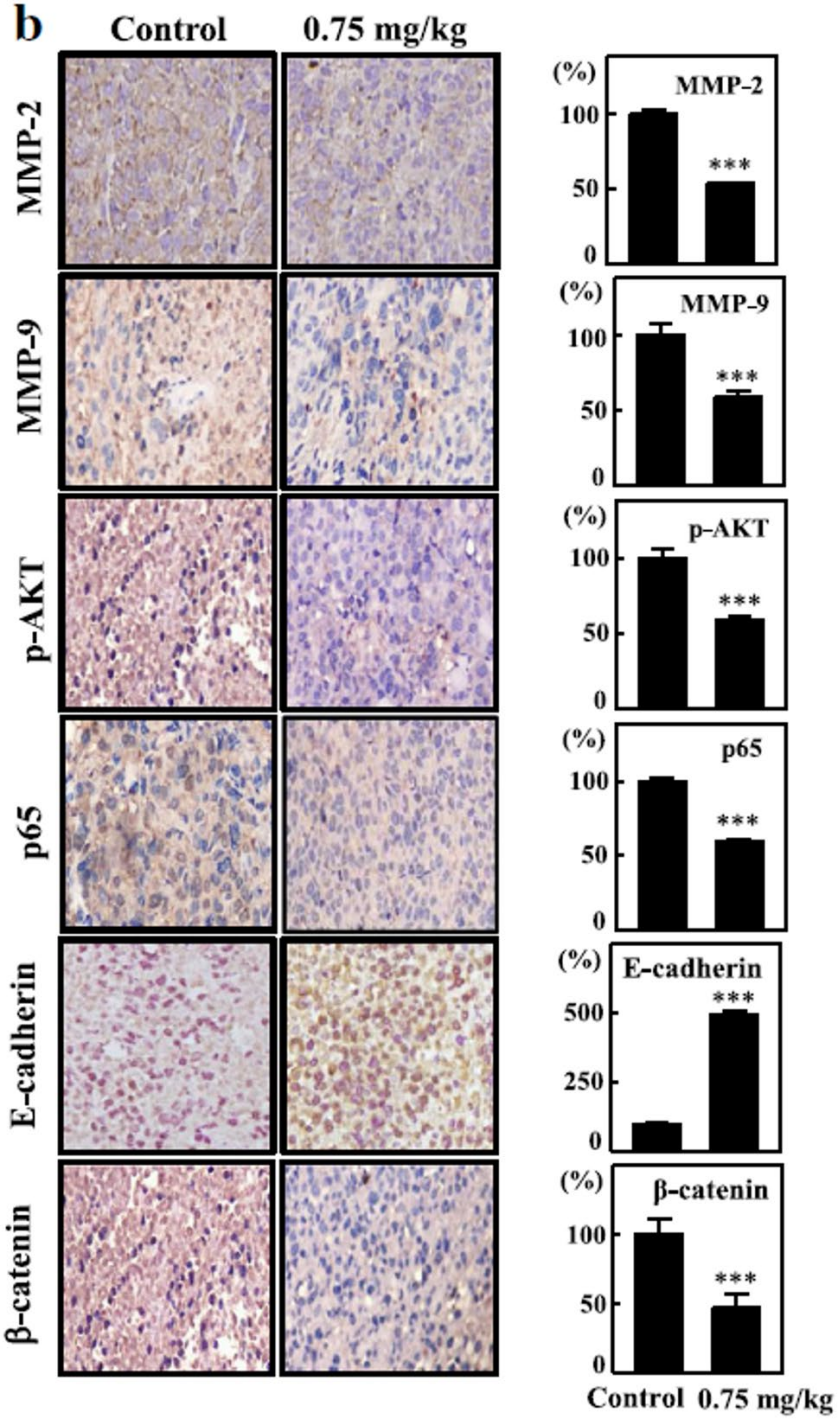
In vivo anti-metastatic activity of CoQ_0_. **a** CoQ_0_ inhibited lung metastasis in living MDA-MB-231-luciferase-injected mice by bioluminescence imaging. Mice were treated with CoQ_0_ (1.5 or 2 mg/kg) and then the MDA-MB-231-luciferase cells (1 × 106 cells/well) were intravenously injected. The mice were anaesthetized, and luciferin was intraperitoneally injected. The mice were imaged using the IVIS 200 system, and the photons from the whole animal were quantified. Diagrams showing the bioluminescent signal emitted from the whole body. The color overlay on the image represents the luminescence (photons/sec) emitted from the animal, as indicated by the color scales. Photos are representative images (*n* = 4). **b-c** Tumor sections were from control animals and experimental analogues treated with CoQ_0_ (0.75 mg/kg). **b** MMP-2, MMP-9, p-AKT, p65, E-cadherin, and β-catenin were examined using immunohistochemical staining. **c** MMP-2, MMP-9, p-AKT, p65, E-cadherin, and β-catenin were examined using Western blotting. The results are the mean (± SE) numbers of cells/microscope field (as percentage) for 3 animals per group. Western blotting results showing the effects of CoQ_0_ on the cumulative protein content in the xenograft tumors. β-actin were used as an internal control. Relative changes in protein bands were measured by densitometric analysis with the control being 100%. The results are the mean (± SE) numbers of cells/microscope field (as percentage) for 5 ~ 7 animals per group. Significant at *p < 0.05; **p < 0.01; ***p < 0.001 compared to untreated control cells

**Correct** Fig. 9b

**Fig. 9:**
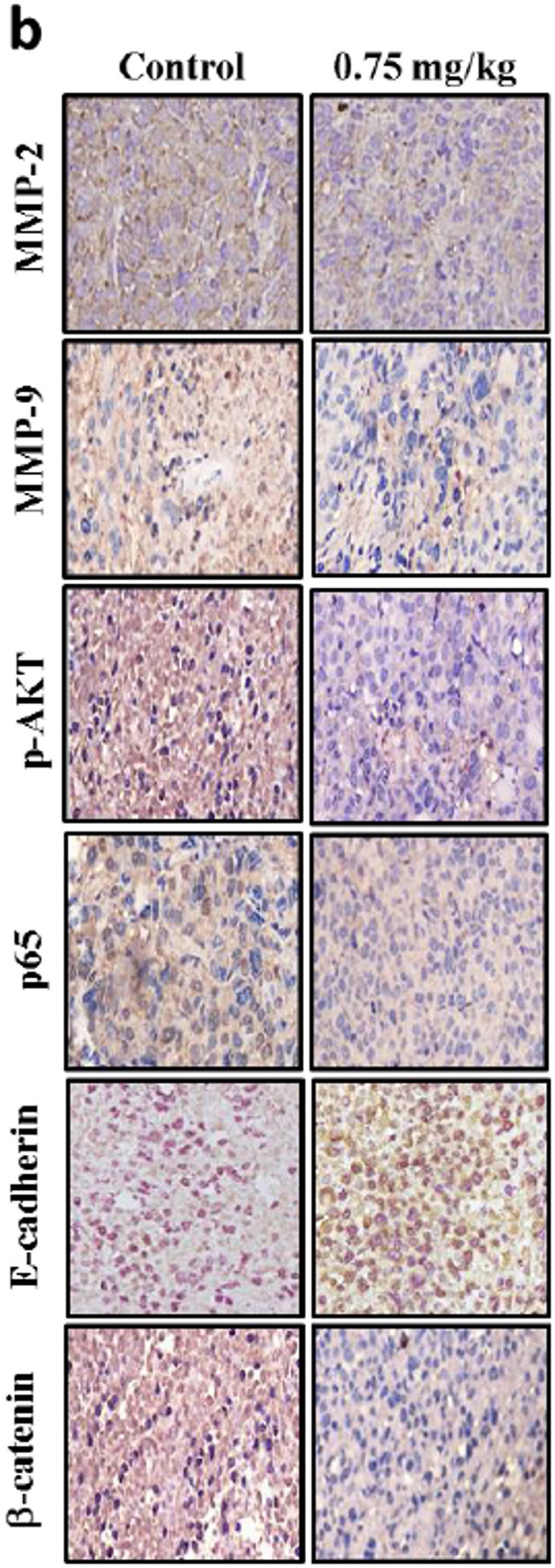
In vivo anti-metastatic activity of CoQ_0_. **a** CoQ_0_ inhibited lung metastasis in living MDA-MB-231-luciferase-injected mice by bioluminescence imaging. Mice were treated with CoQ_0_ (1.5 or 2 mg/kg) and then the MDA-MB-231-luciferase cells (1 × 106 cells/well) were intravenously injected. The mice were anaesthetized, and luciferin was intraperitoneally injected. The mice were imaged using the IVIS 200 system, and the photons from the whole animal were quantified. Diagrams showing the bioluminescent signal emitted from the whole body. The color overlay on the image represents the luminescence (photons/sec) emitted from the animal, as indicated by the color scales. Photos are representative images (*n* = 4). **b-c** Tumor sections were from control animals and experimental analogues treated with CoQ_0_ (0.75 mg/kg). **b** MMP-2, MMP-9, p-AKT, p65, E-cadherin, and β-catenin were examined using immunohistochemical staining. **c** MMP-2, MMP-9, p-AKT, p65, E-cadherin, and β-catenin were examined using Western blotting. The results are the mean (± SE) numbers of cells/microscope field (as percentage) for 3 animals per group. Western blotting results showing the effects of CoQ_0_ on the cumulative protein content in the xenograft tumors. β-actin were used as an internal control. Relative changes in protein bands were measured by densitometric analysis with the control being 100%. The results are the mean (± SE) numbers of cells/microscope field (as percentage) for 5 ~ 7 animals per group. Significant at *p < 0.05; **p < 0.01; ***p < 0.001 compared to untreated control cells
