# Supplementary material for: Anti-EMT properties of CoQ0 attributed to PI3K/AKT/NFKB/MMP-9 signaling pathway through ROS-mediated apoptosis
Source: J Exp Clin Cancer Res. 2019 May 8;38:186. doi: 10.1186/s13046-019-1196-x (PMC6505074; doi:10.1186/s13046-019-1196-x)
Supplement: Supplementary file 1 — CoQ0-induced apoptosis in MDA-MB-231 cells. The cells were exposed to CoQ0 (5–15 μM for 24 h). (a-b) The TUNEL assay was performed to determine apoptotic DNA fragmentation. The green florescence indicates the number of TUNEL positive cells in the microscopic fields (400 × magnification) from three separate samples. The percentage of apoptotic cells was calculated by measuring the florescence intensity of treated cells using commercially available software. (c) Annexin V-FITC and PI staining was used to identify the early/late apoptosis or necrosis, and the data were analyzed using flow cytometry. The results in each quadrant are labeled and interpreted as follows: (Q1) PI positive, Annexin V-FITC-negative stained cells/necrosis. (Q2) PI positive, Annexin V-FITC-positive stained cells/late apoptosis. (Q3) Cells negative for both PI and Annexin V-FITC staining/normal live cells. (Q4) PI-negative, Annexin V-FITC-positive stained cells/early apoptosis. (d) Effects of CoQ0 on apoptotic-related proteins. Protein levels of mitochondria/cytosolic cytochrome c, caspases-9, caspase-3, and PARP, Bax, Bcl-2, and p53 were analyzed by Western blotting. The results are presented as the mean ± SD of three independent assays. ***p < 0.001 significant compared to control cells. (PPTX 51519 kb) [file 13046_2019_1196_MOESM1_ESM.pptx]

## Slide 1
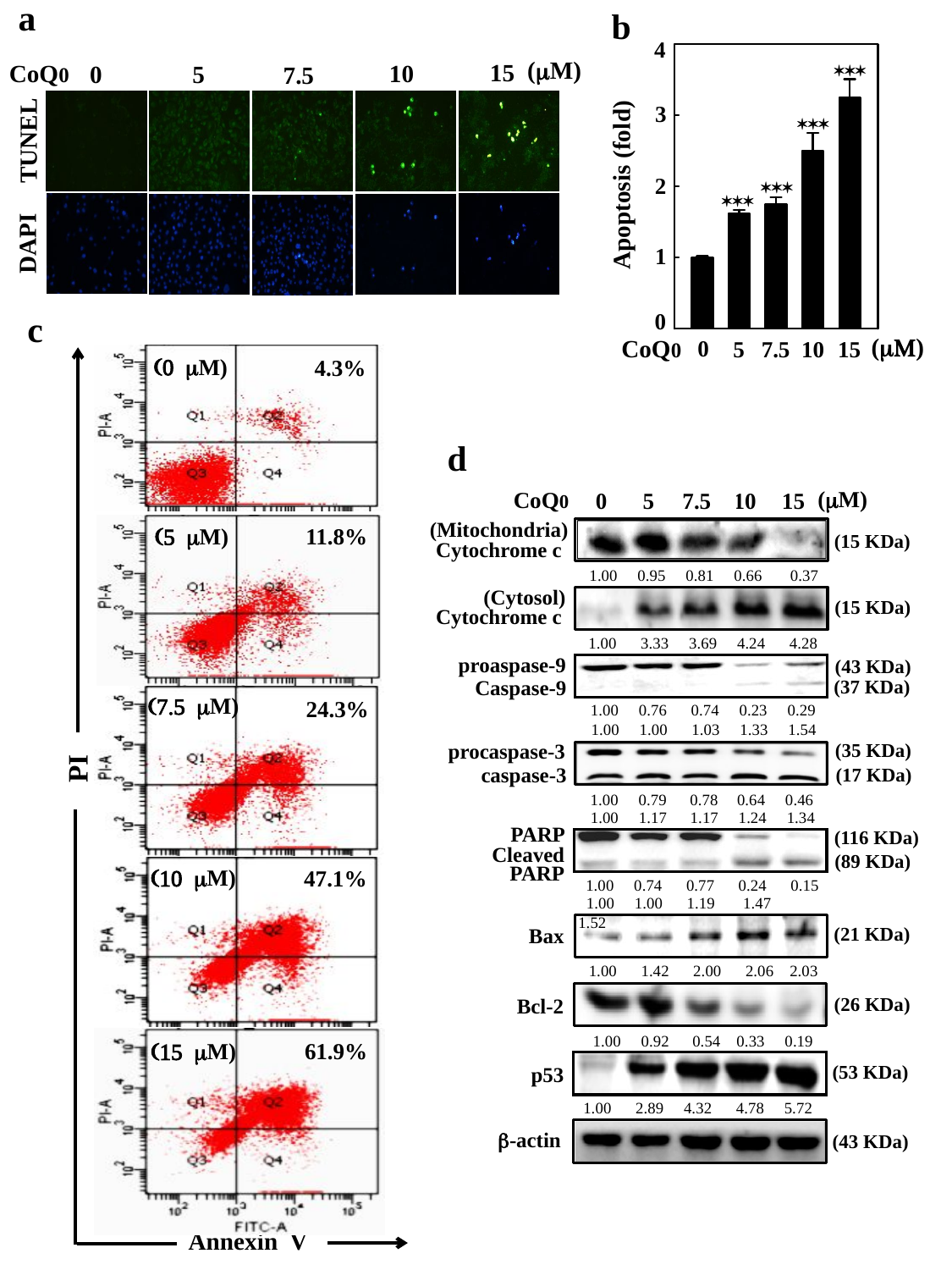

a
(mM)
15
CoQ0
10
5
0
7.5
TUNEL
DAPI
b
4
***
3
***
Apoptosis (fold)
2
***
***
1
0
(mM)
CoQ0
0
5
15
10
7.5
 c
4.3%
(0 mM)
d
(mM)
CoQ0
7.5
0
5
10
15
 (Mitochondria)
(15 KDa)
Cytochrome c
 1.00 0.95 0.81 0.66 0.37
(Cytosol)
(15 KDa)
Cytochrome c
 1.00 3.33 3.69 4.24 4.28
proaspase-9
(43 KDa)
(37 KDa)
Caspase-9
 1.00 0.76 0.74 0.23 0.29
1.00 1.00 1.03 1.33 1.54
procaspase-3
(35 KDa)
(17 KDa)
caspase-3
 1.00 0.79 0.78 0.64 0.46
 1.00 1.17 1.17 1.24 1.34
PARP
(116 KDa)
Cleaved
PARP
(89 KDa)
1.00 0.74 0.77 0.24 0.15
 1.00 1.00 1.19 1.47 1.52
(21 KDa)
Bax
 1.00 1.42 2.00 2.06 2.03
Bcl-2
(26 KDa)
1.00 0.92 0.54 0.33 0.19
(53 KDa)
p53
 1.00 2.89 4.32 4.78 5.72
b-actin
(43 KDa)
11.8%
(5 mM)
24.3%
(7.5 mM)
PI
47.1%
(10 mM)
61.9%
(15 mM)
Annexin V
