# Supplementary material for: Anti-EMT properties of CoQ0 attributed to PI3K/AKT/NFKB/MMP-9 signaling pathway through ROS-mediated apoptosis
Source: J Exp Clin Cancer Res. 2019 May 8;38:186. doi: 10.1186/s13046-019-1196-x (PMC6505074; doi:10.1186/s13046-019-1196-x)
Supplement: Supplementary file 2 — CoQ0 treatment induces G2/ M cell-cycle arrest in MDA-MB-231 cells. (a) Cells were treated with CoQ0 (5–15 μM) for 24 h, stained with PI and analyzed for cell-cycle phase using flow cytometry. The cellular distributions (%) in different phases of the cell cycle (sub-G1, G1,S and G2/M) were determined after treatment with AS. The flow cytometry graph shown here is from one representative experiment that was performed in triplicates. (b) The effects of CoQ0 AS on cell-cycle regulatory proteins. HL-60 cells were treated with increasing concentrations of CoQ0 (5–15 μM) for 24 h. Cell-cycle regulatory proteins, including Cyclin A, Cyclin B, p21, Cdc2, Cdc25C, CDK2, and CDK4 were examined using Western blot analyses. The results are presented as the mean ± SD of three independent assays. **p < 0.05, ***p < 0.001 significant compared to control cells. (PPTX 4253 kb) [file 13046_2019_1196_MOESM2_ESM.pptx]

## Slide 1
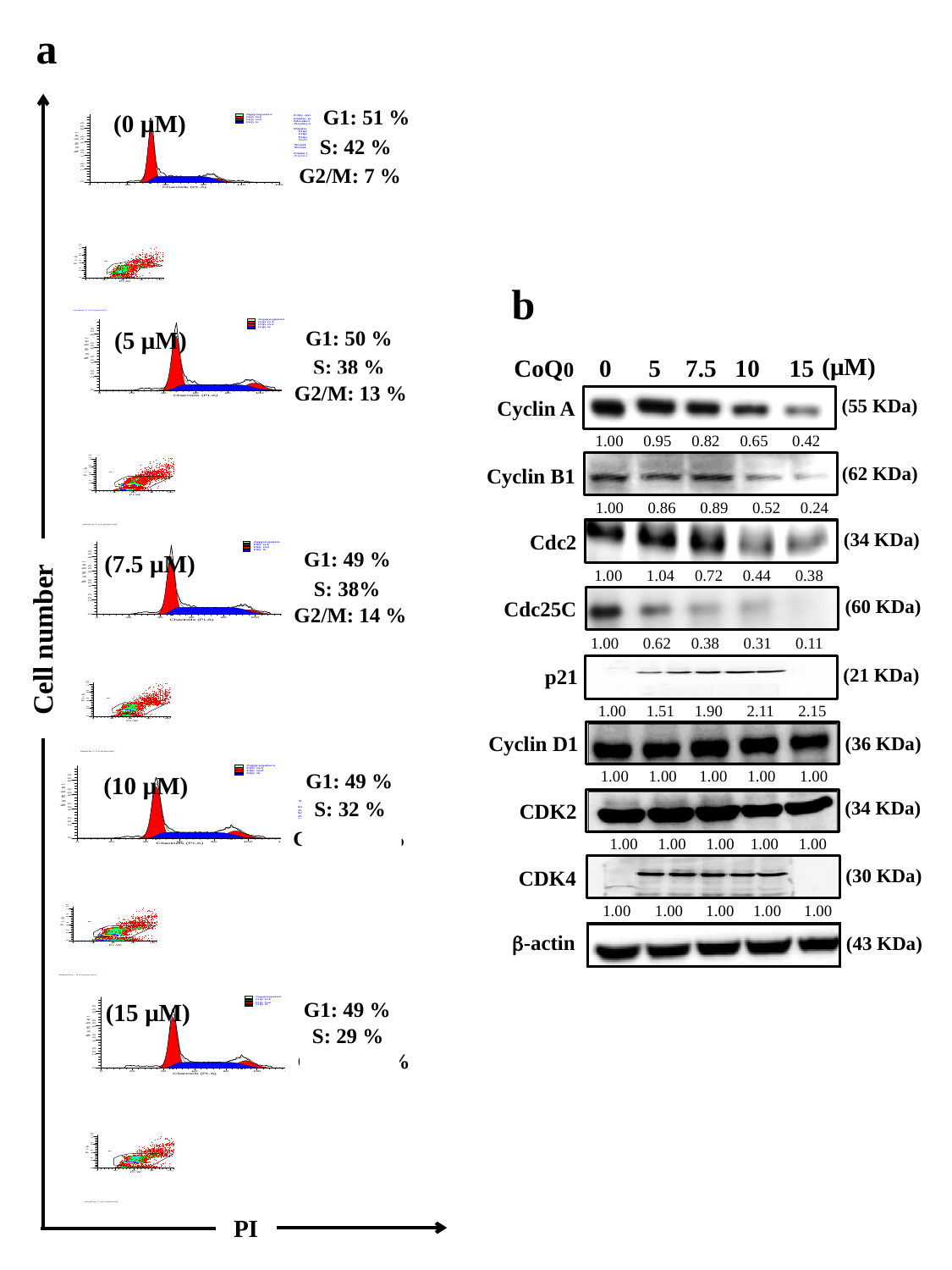

a
G1: 51 %
S: 42 %
G2/M: 7 %
(0 μM)
G1: 50 %
S: 38 %
G2/M: 13 %
(5 μM)
G1: 49 %
S: 38%
G2/M: 14 %
(7.5 μM)
Cell number
G1: 49 %
S: 32 %
G2/M: 19 %
(10 μM)
G1: 49 %
S: 29 %
G2/M: 22 %
(15 μM)
PI
 b
(μM)
CoQ0
 0 5 7.5 10 15
(55 KDa)
Cyclin A
1.00 0.95 0.82 0.65 0.42
(62 KDa)
Cyclin B1
1.00 0.86 0.89 0.52 0.24
(34 KDa)
Cdc2
1.00 1.04 0.72 0.44 0.38
(60 KDa)
Cdc25C
1.00 0.62 0.38 0.31 0.11
(21 KDa)
p21
1.00 1.51 1.90 2.11 2.15
Cyclin D1
(36 KDa)
1.00 1.00 1.00 1.00 1.00
(34 KDa)
CDK2
1.00 1.00 1.00 1.00 1.00
(30 KDa)
CDK4
1.00 1.00 1.00 1.00 1.00
1.0 1.5 1.8 2.1 2.1
b-actin
(43 KDa)
