# Supplementary figures and images for: Anti-EMT properties of CoQ0 attributed to PI3K/AKT/NFKB/MMP-9 signaling pathway through ROS-mediated apoptosis
Source: J Exp Clin Cancer Res. 2019 May 8;38:186. doi: 10.1186/s13046-019-1196-x (PMC6505074; doi:10.1186/s13046-019-1196-x)

## Slide 1
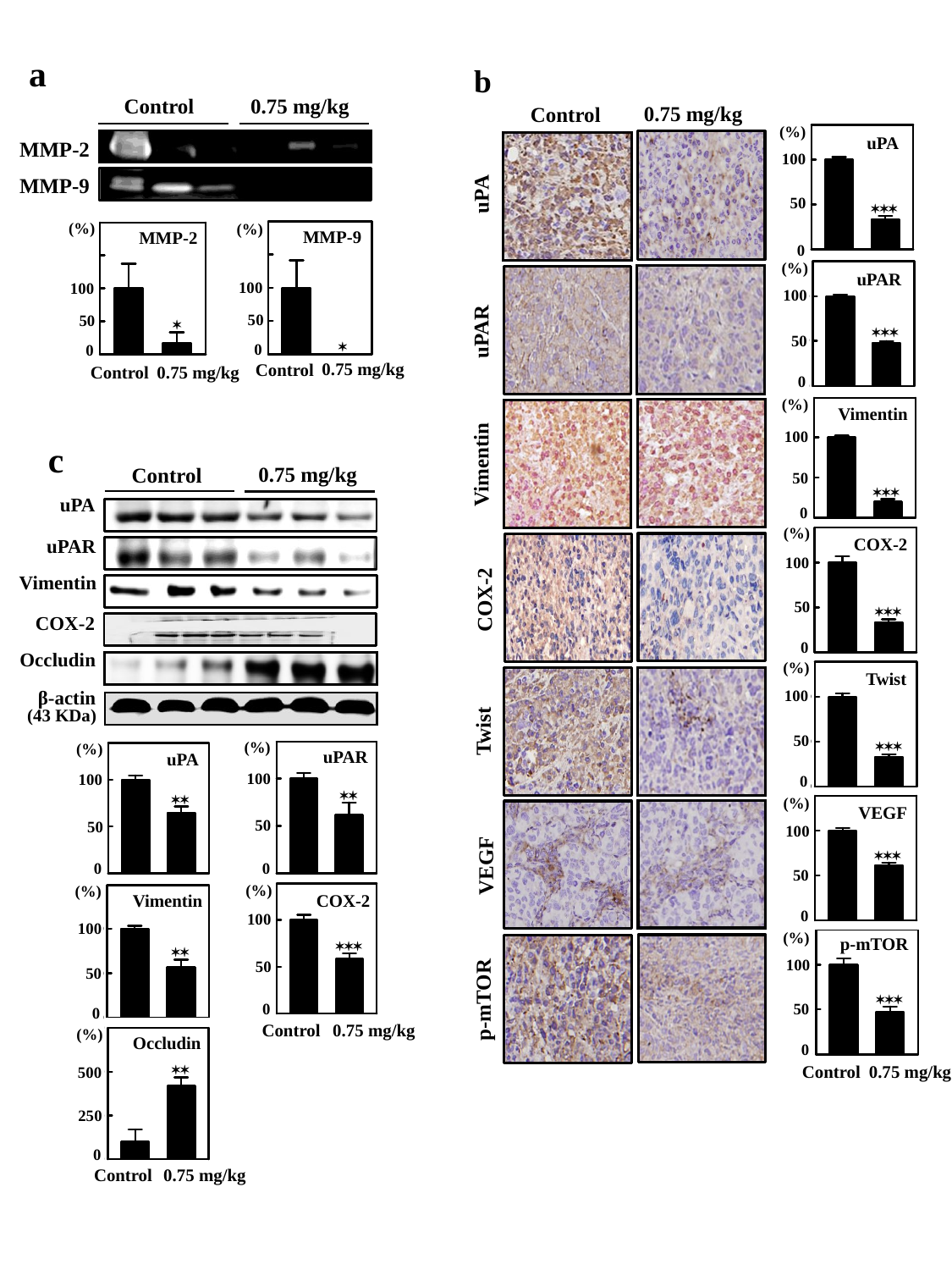

Supplement: Supplementary file 3 — Metastasis and EMT inhibition by CoQ0 in MDA-MB-231 xenografted tumors. Tumor sections were from control animals and experimental analogues treated with CoQ0 (0.75 mg/kg). (a) Cells positive for the indicated proteins were counted from 3 fields (200 × magnification) for each tumor sample, and MMP-2 and MMP-9 were examined using RT-PCR. (b) uPA, uPAR, Vimentin, COX-2, Twist, VEGF, and p-mTOR were examined using immunohistochemical staining. (c) uPA, uPAR, Vimentin, COX-2, and Occludin were examined using Western blotting. Western blot on the effects of CoQ0 on the total protein contents in the xenograft tumors. β-actin was used as the control. Relative changes in protein bands were measured by densitometric analysis with the control being 100%. The results are the mean (±SE) numbers of cells/microscope field (as percentage) for 3 animals per group. Significant at *p < 0.05; **p < 0.01; ***p < 0.001 compared to untreated control cells. (PPTX 12155 kb) [file 13046_2019_1196_MOESM3_ESM.pptx]
